# A ^1^H NMR-Based Metabonomic Investigation of Time-Related Metabolic Trajectories of the Plasma, Urine and Liver Extracts of Hyperlipidemic Hamsters

**DOI:** 10.1371/journal.pone.0066786

**Published:** 2013-06-26

**Authors:** Chun-ying Jiang, Kang-min Yang, Liu Yang, Zhao-xia Miao, Ying-hong Wang, Hai-bo Zhu

**Affiliations:** State Key Laboratory of Bioactive Substance and Function of Natural Medicines & Ministry of Health Key Laboratory of Biosynthesis of Natural Products, Chinese Academy of Medical Sciences and Peking Union Medical College, Beijing, China; Spanish National Cancer Center, Spain

## Abstract

The hamster has been previously found to be a suitable model to study the changes associated with diet-induced hyperlipidemia in humans. Traditionally, studies of hyperlipidemia utilize serum- or plasma-based biochemical assays and histopathological evaluation. However, unbiased metabonomic technologies have the potential to identify novel biomarkers of disease. Thus, to obtain a better understanding of the progression of hyperlipidemia and discover potential biomarkers, we have used a proton nuclear magnetic resonance spectroscopy (^1^H-NMR)-based metabonomics approach to study the metabolic changes occurring in the plasma, urine and liver extracts of hamsters fed a high-fat/high-cholesterol diet. Samples were collected at different time points during the progression of hyperlipidemia, and individual proton NMR spectra were visually and statistically assessed using two multivariate analyses (MVA): principal component analysis (PCA) and orthogonal partial least squares-discriminant analysis (OPLS-DA). Using the commercial software package Chenomx NMR suite, 40 endogenous metabolites in the plasma, 80 in the urine and 60 in the water-soluble fraction of liver extracts were quantified. NMR analysis of all samples showed a time-dependent transition from a physiological to a pathophysiological state during the progression of hyperlipidemia. Analysis of the identified biomarkers of hyperlipidemia suggests that significant perturbations of lipid and amino acid metabolism, as well as inflammation, oxidative stress and changes in gut microbiota metabolites, occurred following cholesterol overloading. The results of this study substantially broaden the metabonomic coverage of hyperlipidemia, enhance our understanding of the mechanism of hyperlipidemia and demonstrate the effectiveness of the NMR-based metabonomics approach to study a complex disease.

## Introduction

Hyperlipidemia is defined as the presence of abnormally elevated levels of lipids and/or lipoproteins in the plasma (involving hypertriglyceridemia, hypercholesterolemia or both), and can raise the risks of coronary heart disease, fatty liver disease, and cancer [1]. Dietary fat is presently regarded as an important environmental factor associated with the incidence of this metabolic syndrome. Improved understanding of the pathophysiology of hyperlipidemia is necessary for better prevention and treatment of this disease.

Studies of hyperlipidemia commonly utilize serum- or plasma-based biochemical assays and histopathological evaluation. However, these standard approaches may be inadequate. Metabonomics, with its impressive and ever-increasing coverage of endogenous compounds, as well as its intrinsic high-throughput capacity, has been demonstrated to be a valuable approach to study the complex biological responses to chemical and physical perturbations at the metabolic level. It offers an unbiased view of the pathological conditions and also generates diagnostic information [2], [3]. The metabonomic approach has shown particular promise in investigating the cardiovascular disease progression and the mechanisms by which diet impacts cardiovascular health. For example, a proton nuclear magnetic resonance spectroscopy (^1^H-NMR)-based metabonomic approach was utilized to study the progression of coronary atherosclerosis in a rabbit model [4]. In animal models of atherosclerosis, it was shown that certain plasma and urine metabolites were altered upon development of the disease [5], and time-dependent changes during the progression from a healthy state to hypercholesterolemia and early atherosclerosis were observed [6]. The amount of milk fat in the diet was shown to correlate positively with the degree of atherogenicity in hyperlipidemic hamsters [7], while plant sterol esters (enriched with stearate) may lower the level of low-density lipoprotein cholesterol (LDL-C) in humans [8]. Consumption of whole-grain rye versus non-whole-grain wheat diets was found to yield major differences in the plasma metabolome of hypercholesterolemic pigs, particularly with regards to betaine levels [9]. In our previous study, we used ^1^H-NMR-based metabonomics to evaluate the beneficial effects of 29,39,59-tri-acetyl-N6-(3-hydroxylaniline) adenosine (WS070117) for hyperlipidemic Syrian golden hamsters [10]. However, to the best of our knowledge, there has been no systematic investigation of the progression of hyperlipidemia using a quantitative metabolomic approach. “Quantitative” analysis of metabolites may significantly improve our ability to identify disease-related biomarkers. This approach involves the quantification of a large number of metabolites, whose characteristics (e.g., NMR spectra) are known and stored in a database library, present in a single specimen in a high-throughput manner [11].

In this study, we have investigated the progression of hyperlipidemia in a hamster model using ^1^H-NMR-based metabolomics, including both the traditional metabolomic approach and quantitative metabolomics coupled with multivariate data analysis. Our findings provide novel insights into the metabolic changes occurring during progression of hyperlipidemia.

## Materials and Methods

### Experimental Design

#### Ethics statement

The experiments were approved by the Research Ethics Committee of the Chinese Academy of Medical Sciences and Peking Union Medical College (approval number: PUM201033334A). Animals were maintained and experiments were conducted in accordance with the Institutional Animal Care and Use Committee, Chinese Academy of Medical Sciences and Peking Union Medical College, and with the 1996 Guide for the Care and Use of Laboratory Animals (Institute of Laboratory Animal Resources on Life Sciences, National Research Council, National Academy of Sciences, Washington DC).

#### Subjects

Twelve-week-old Syrian golden hamsters were obtained from Vital River Laboratory Animal Technology Co. Ltd. (Beijing, China). Throughout the acclimatization and study periods, all animals had ad libitum access to food and water and were maintained on a 12-h light/dark cycle in a facility with an ambient temperature of 21±2°C and a relative humidity of 45±10%. One hundred and eight adult male Syrian golden hamsters (90–110 g) were acclimatized for 7 days in cages prior to the initiation of the study. The animals were then randomly allocated into three groups according to the length of the experiment: short-term group (4 weeks), middle-term group (24 weeks) and long-term group (42 weeks). Animals in each group were then randomly allocated into two subgroups, control (n = 16) and HFHC (n = 20). The hamsters that served as the reference group were fed the standard chow ad libitum, while the hyperlipidemic hamsters were fed a high-fat, high-cholesterol (HFHC) diet (Institute of Laboratory Animal Sciences, Beijing, China) daily to establish the hamster model of hyperlipidemia. The standard chow was composed of 98% standard diet and 2% fat, while the HFHC diet was composed of 85.2% standard diet, 14.5% fat, and 0.3% cholesterol.

### Measurement of Lipid Content in Serum

Hamsters were anesthetized with an intraperitoneal injection of 3% sodium pentobarbital (2 ml per kg). An abdominal incision was made to expose the liver and inferior vena cava. Blood (3–4 ml) was withdrawn from the abdominal aorta and collected in tubes with or without heparin in order to collect the plasma and serum, respectively. The serum levels of total cholesterol (TC), triglyceride (TG), low-density lipoprotein cholesterol (LDL-C), and high-density lipoprotein cholesterol (HDL-C) were measured using commercial enzymatic assays (BioSino Biotechnology and Science Inc). Each sample was assayed in duplicate.

### Oil Red O and H&E Staining

Sections of liver tissue were fixed in 4% paraformaldehyde and dehydrated in a 30% sucrose solution at room temperature. Tissues were then immersed in Optimal Cutting Temperature (OCT) solution on dry ice. Three different tissue sections from each liver were processed and stained using routine laboratory procedures. Formalin-fixed and paraffin-embedded livers were routinely processed for hematoxylin and eosin (H&E) staining.

### Ultrasonographic Imaging

Following an overnight fasting, the animals were anesthetized by inhalation of 1.5%–2% isoflurane gas. After removing hair from the abdomen and anchoring the limbs to a constant temperature circuit platform in a supine position, the livers of the animals were examined using an ultra imager (VisualSonics Vevo 770H High-Resolution Imaging System) equipped with a real-time micro-visualization scan-head probe.

### ^1^H-NMR Spectroscopy of Plasma, Urine and Liver Extract Samples

#### Metabolite sample preparation

Thirty microliters of plasma was added to 60 µL of 0.9% saline (D_2_O:H_2_O = 1∶9) containing 0.1% sodium 3-trimethylsilyl-propionate-2, 2, 3, 3,-d4 (TSP) (an internal standard, chemical shift δ 0.0 ppm) in Eppendorf tubes. Samples were centrifuged at 3,000 g for 5 min at 278 K, and 60 µL of sample was transferred into 1.7-mm NMR tubes. A first increment of NOESY pulse sequence [recycle delay-90°-t_1_-90°-t_m_-90°-acquisition], CPMG (Carr-Purcell-Meiboom-Gill) and BPP-LED (bipolar pulse pair-longitudinal eddy current delay) spectra were recorded. In addition, 300 µL of plasma was applied to 3 kDa Nanosep microcentrifuge filters to remove proteins and insoluble impurities. Following this step, 10 µL of 0.1% sodium buffer containing dimethyl-silapentane-sulfonate (DSS; final concentration, 0.5 mM) was added to 90 µL of filtrate. A 180 µL aliquot of urine was diluted with 20 µL of buffer solution (0.2 M Na_2_HPO4/0.2 M NaH_2_PO_4_, pH 7.4) containing DSS (final concentration, 0.5 mM) and then centrifuged at 13,000 g for 5 min to remove particulate matter. Then, 60 µL of plasma or urine sample was transferred into 1.7-mm NMR tubes. Hamster liver samples were prepared using the method described by Atherton et al. [12] with slight modifications. Briefly, liver samples (about 100 mg) were homogenized for 1 min in ice-cold methanol/chloroform (2∶1, v/v, 3 mL) using a Heidolph Diax 600 homogenizer (Schwabach, Germany). Samples were then sonicated for 30 min and diluted with 1 mL of ice-cold water and 1 mL of ice-cold chloroform. This mixture was then vortexed for 1 min, followed by centrifugation at 4000 g for 20 min. The aqueous supernatant (polar phase) was then collected and dried in a freeze dryer(FDU-1100; EYELA, Tokyo Rikakikai CO., LTD.; Japan) to obtain the water-soluble fraction of liver extracts, while the organic phase was dried in a nitrogen evaporator to obtain the lipid extracts. The water-soluble phase of liver extracts was dissolved in D_2_O containing dimethyl-silapentane-sulfonate (DSS, final concentration, 0.5 mM), and lipid extracts were dissolved in chloroform-d containing tetramethylsilane (TMS). In these experiments, DSS, an NMR chemical shift reference compound, was employed for concentration calibration for NMR chemical shift reference and concentration calibration. One-dimensional spectra were acquired using a first increment of NOESY pulse sequence mentioned above.

#### NMR spectra acquisition

NMR spectra were acquired using an automated NMR Case sample changer on a Bruker Avance 500 spectrometer (Bruker Biospin) operating at 500.13 MHz and equipped with a 1.7-mm TXI probe at 298 K. Three kinds of ^1^H NMR spectra were acquired: a standard one-dimensional pulse sequence using the first increment of the NOESY pulse sequence to achieve water pre-saturation, a Carr-Purcell-Meiboom-Gill (CPMG) pulse sequence [13] to enhance the contribution of low molecular weight metabolites, and a diffusion-edited experiment using a bipolar pulse pair-longitudinal eddy current delay (BPP-LED) pulse sequence [13]–[15]to measure the lipid content of plasma lipoproteins. For the standard one-dimensional experiment, the mixing time (t_m_) was 100 ms. For the CPMG experiment, a spin-spin relaxation delay of 320 ms was used for each sample, and water signal irradiation was applied during the recycle delay. For the BPP-LED experiment, a sine-shaped gradient with a strength of 32 G/cm and a duration of 2.5 ms was followed by a delay of 400 ms to allow for the decay of eddy currents. A diffusion delay of 120 ms and a delay T_e_ of 5 ms were used. A line-broadening factor of 0.3–1 Hz was applied to FIDs before Fourier transformation. Spectra were acquired with 128 scans, then zero filled and Fourier-transformed to 128 k data points. For proper quantitative fitting of the NMR spectra, it is important that the spectra are collected under the same conditions as the metabolite standard spectra in the Chenomx database. Additional 2-dimensional NMR experiments were performed for the purpose of confirming chemical shift assignments, including homonuclear total correlation spectroscopy (2D ^1^H-^1^H TOCSY) and heteronuclear single quantum coherence spectroscopy (2D ^1^H-^13^C HSQC), using standard Bruker pulse programs.

#### Data analysis

^1^H-NMR spectra were manually corrected for phase and baseline distortions using TOPSPIN (version 3.0, Bruker Biospin) and referenced to the TSP signal (δ 0.0). The ^1^H NMR spectra of plasma specimens were binned into 0.04 ppm integral regions and integrated in the region 0.5–6.0 ppm using the AMIX software package (version 3.8.3, Bruker Biospin). The regions containing the water resonance (δ 5.1−4.7) were removed. The ^1^H-NMR spectra obtained from the chloroform phase of liver extracts were binned into 0.04 ppm integral regions and integrated in the region 0.3–6.0 ppm. The spectra were normalized to the total sum of the spectral integrals to compensate for differences in sample concentration. The multivariate data analyses of the normalized NMR data sets were carried out using the SIMCA-P^+^ software package (version 12.0, Umetrics; Sweden).

For quantitative metabolomic profiling of plasma, urine and the water-soluble fraction of liver extracts, processed spectra were imported into the Chenomx NMR Suite 7.5 software (Chenomx Inc., Edmonton, Canada) and metabolites were quantified using the ‘targeted profiling’ approach, where individual NMR resonances of interest were mathematically modeled from pure standard metabolite compound spectra stored in an internal database, and this database was then interrogated to identify and quantify metabolites present in the complex spectra of biofluids. Overall, we detected 80 compounds in urine, 40 compounds in plasma and 60 compounds in the water-soluble fraction of liver homogenates with sufficient signal-to-noise ratios. Spectra were randomly ordered for profiling. Compounds were profiled in order of decreasing typical concentration. Each compound concentration was then normalized to the total concentration of all metabolites in the sample (with the exclusion of urea for urine samples, as its excessively high concentration would have otherwise distorted the normalization).

To reveal shifts in metabolite concentration, multivariate analysis was conducted using SIMCA-P^+^12.0 software (Umetrics; Sweden). Initially, the principal component analysis (PCA) of the NMR spectral data was performed (on mean-centered data) to visualize the general structure of each data set and to identify any abnormalities within the data set. Subsequently, a supervised multivariate data analytical tool, orthogonal projection to latent structure discriminant analysis (OPLS-DA), was applied to the analysis of ^1^H NMR spectral data scaled to unit variance [16], [17]. To check the validity of the model and avoid the overfitting of the PLS model, the assessment of the 7-fold cross-validated scores from the model was used and the cross-validation parameter Q^2^, indicating the predictability of the model related to its statistical validity, was calculated [18]. An additional cross validation tool, a permutation test, was performed for each model by randomizing the order of Y variables for a specified number of times (permutation number = 200). The R^2^ in the permutated plot describes how well the data fit with the derived model, whereas Q^2^ describes the predictive ability of the derived model and provides a measure of the model quality with Q^2^>0.5 considered as ‘good’ and Q^2^>0.9 considered as ‘excellent’. If higher Q^2^ values were obtained from the permutation models than the one from the true model, then the model was deemed to lack predictive ability. The fact that both Q^2^Y and R^2^Y are close to 1 indicates an excellent model, whereas low values are indicative of model overfitting.

## Results

### Serum Biochemical Parameters

Table 1 shows the relative fold changes in hyperlipidemia parameters of the HFHC group as compared with the control group over the entire study period. Significantly elevated plasma levels of total cholesterol (TC), triglyceride (TG) and low-density lipoprotein cholesterol (LDL-C) were observed in hamsters of the hyperlipidemia group as compared with the healthy control group over the entire study period.

**Table 1:** Pathophysiological changes in HFHC-fed hyperlipidemic hamsters at the different time points.

Parameters	Fold change^d^		
	Week 6	Week 15	Week 21
TG (mmol/L^−1^)	2.92^b^	6.37^a^	2.29^a^
TC (mmol/L^−1^)	2.23^c^	2.7^b^	1.81^c^
HDL-C (mmol/L^−1^)	1.73^c^	1.85^a^	1.43^c^
LDL-C (mmol/L^−1^)	4.06^c^	3.48^b^	1.63^c^

a p<0.05 as compared with control group.

b p<0.01 as compared with control group.

c p<0.001 as compared with control group.

d relative to the control. Control anmials, n = 16; HFHC animals, n = 20. Statistical analysis was performed using a two-tailed Student’s t-test.

### Ultrasonographic Imaging

**Figure 1:**
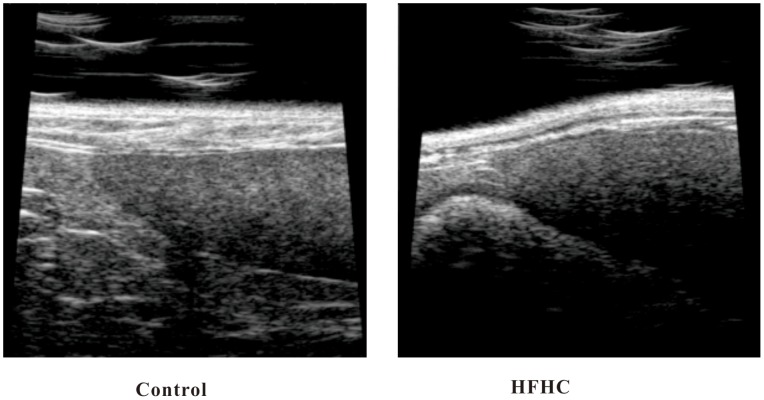
Ultrasonographic imaging of liver tissue from control (left) and HFHC hamsters (right) at week 42.

Fat accumulation in liver causes increased echogenicity, and the liver thus appears smoother and brighter. The obvious increased echogenicity of livers taken from HFHC hamsters is indicative of steatosis in these hamsters (Fig. 1).

### Oil Red O and H&E Staining

**Figure 2:**
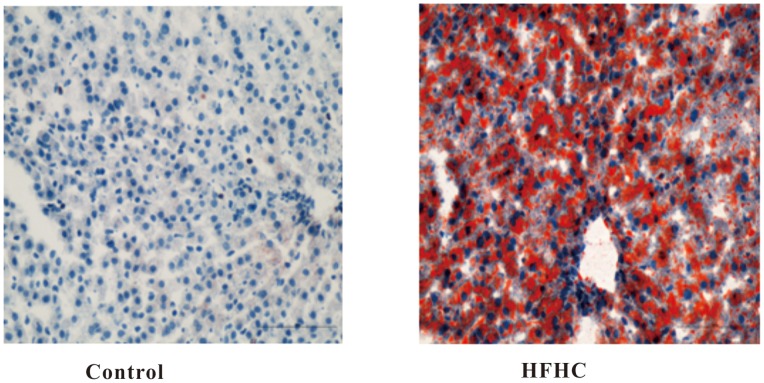
Oil Red O staining of liver tissue from control (left) and HFHC hamsters (right) at week 42 revealed the accumulation of fat in the latter group.

**Figure 3:**
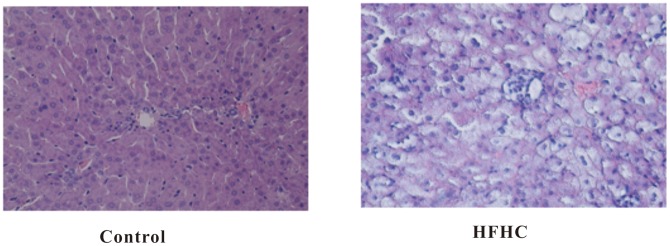
Representative hematoxylin and eosin (H&E) staining of liver sections from control (left) and HFHC hamsters (right) at week 42. Lipid droplets were obviously accumulated in HFHC hamsters, but not control hamsters.

Oil Red O staining was performed to assess hepatic fat content. The hepatocytes of HFHC-fed hyperlipidemic hamsters were found to be compressed. It was observed that the hepatocytes were separated by bulks of fat, and the livers were of a strikingly pale yellow color, indicating an abnormally high level of fat accumulation and deposition (Fig. 2). Accordingly, histologic staining of liver tissue with H&E staining also showed obvious fat droplet accumulation in livers of HFHC-feeding hamsters (Fig. 3).

### ^1^H-NMR Spectroscopic Analysis of Plasma, Urine and Liver Extract Samples

**Figure 4:**
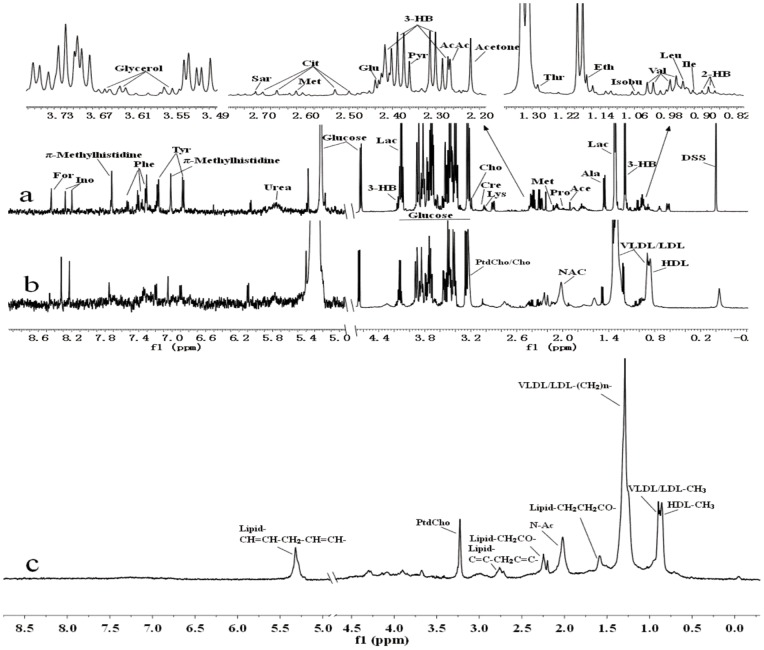
500 MHz ^1^H NMR spectra of plasma samples. (a) ^1^H-NMR spectra of plasma samples after filtration. (b) ^1^H-NMR CPMG spectra of plasma samples. (c) ^1^H-NMR BPP-LED spectra of plasma samples. Abbreviations: 2-HB, 2-hydroxybutyrate; Isobu, isobutyrate; Ile, isoleucine; Leu, leucine; Val, valine; Eth, ethanol; 3-HB, 3-hydroxybutyrate; Lac, lactate; Ala, alanine; Ace, acetate; AcAc, acetoacetate; Pro, proline; Pyr, pyruvate; Glu, glutamine; Cir, citrate; Met, methionine; Sar, sarcosine; Lys, lysine; Cre, creatine; Cho, choline; Tyr, tyrosine; Phe, phenylalanine; Ino, inosine; For, formate; VLDL, very low-density lipoprotein; LDL, low-density lipoprotein; HDL, high-density lipoprotein; PtdCho, phosphatidylcholine; and N-Ac, N-acetyl glycoproteins.

**Figure 5:**
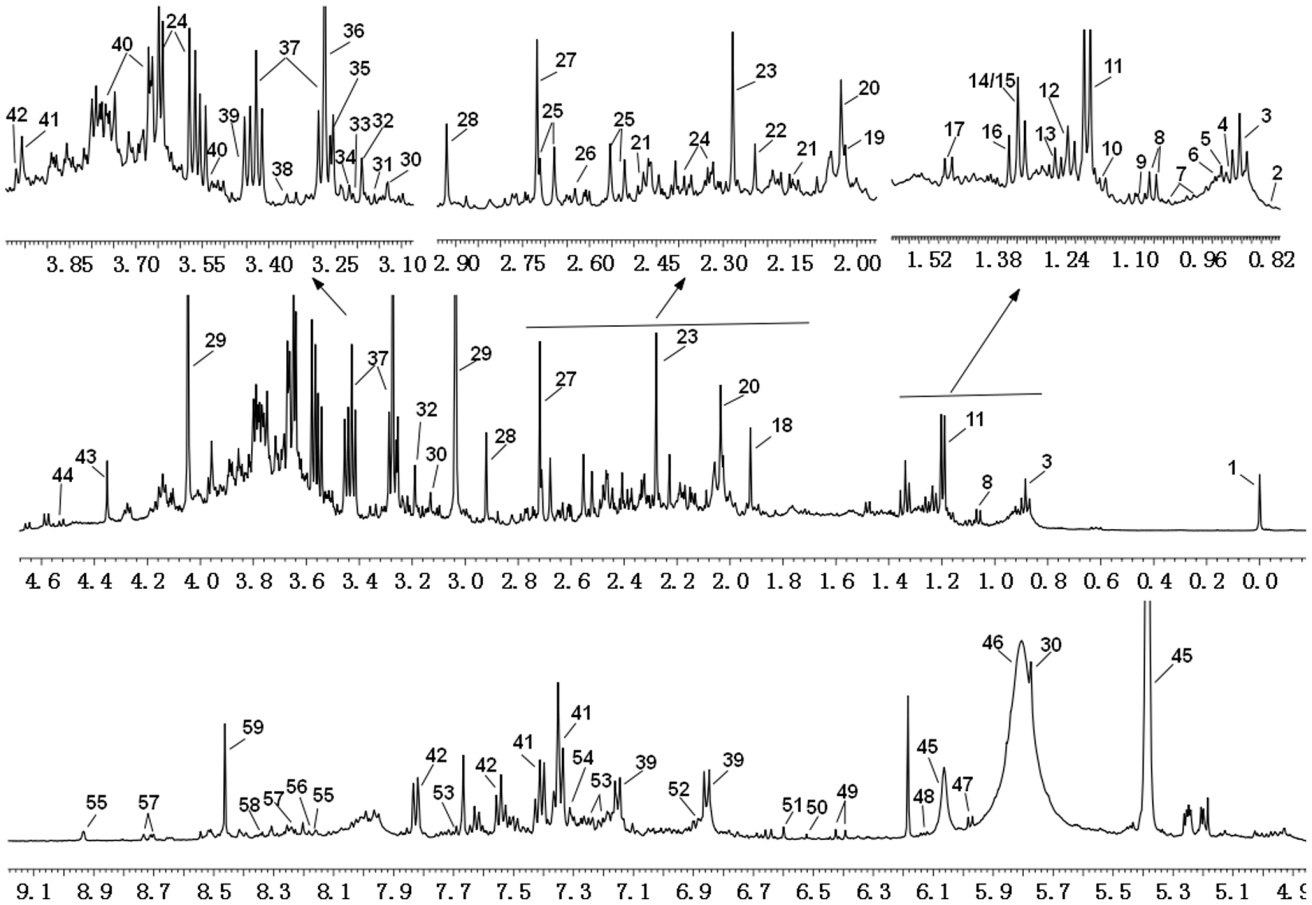
500 MHz ^1^H NMR spectra of urine samples. 1, DSS; 2, 2-Hydroxyisovalerate; 3, 2-Hydroxybutyrate; 4, 2-Hydroxyvalerate; 5, 2-Oxoglutarate; 6, Isoleucine; 7, Valine;8, Isobutyrate; 9, Methylsuccinate; 10, Ethanol; 11, 3-Hydroxybutyrate; 12, Fucose; 13, 3-Hydroxyisovalerate; 14, Lactate; 15, 3-Hydroxy-3-methylglutarate; 16, 2-Hydroxyisobutyrate; 17, Alanine; 18, Acetate; 19, N-Acetylglutamate; 20, N-Acetylglycine; 21, Glutamine; 22, Acetone; 23, Acetoacetate; 24, Glycerol; 25, Citrate; 26, Methylamine; 27, Dimethylamine; 28, N,N-Dimethylglycine; 29, Creatinine; 30, cis-Aconitate; 31, O-Acetylcarnitine; 32, Choline; 33, O-Phosphocholine; 34, Carnitine; 35, Betaine; 36, Trimethylamine N-oxide; 37, Taurine; 38, Methanol; 39, 4-Hydroxyphenylactate; 40, 3-Methylxanthine; 41, N-Phenylacetylglycine; 42, Hippurate; 43, Tartrate; 44, Xylose; 45, Allantoin; 46, Urea; 47, Cytosine; 48, ADP; 49, Urocanate; 50, Fumarate; 51, trans-Aconitate; 52, Tyrosine; 53, 3-Indoxylsulfate; 54, 3-Phenyllactate; 55, Nicotinamide N-oxide; 56, Hypoxanthine; 57, Nicotinurate; 58, Inosine; 59, Formate.

**Figure 6:**
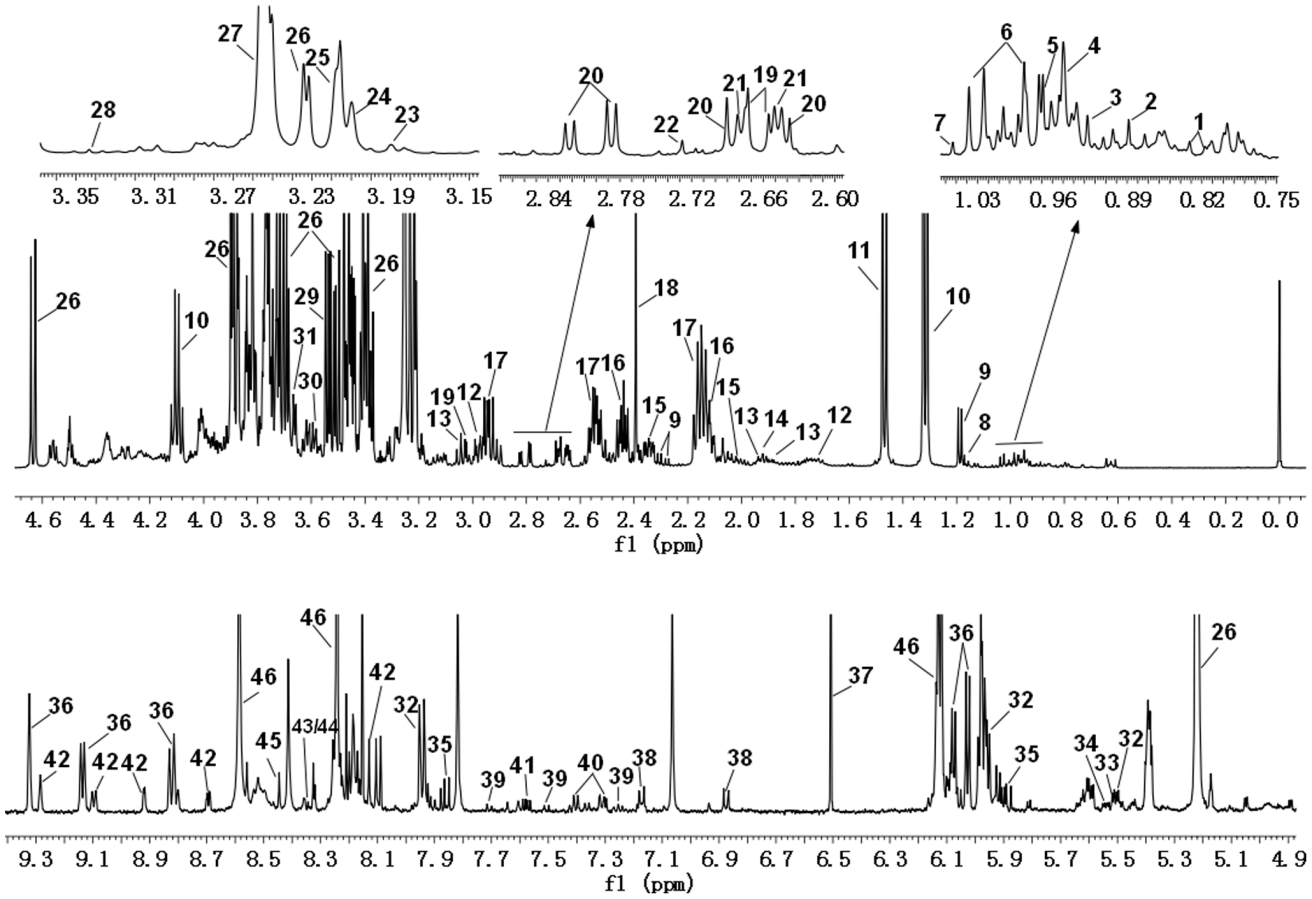
500 MHz ^1^H NMR spectra of aqueous liver extract. 1, 2-Hydroxyisovalerate; 2, 2-Hydroxybutyrate; 3, Isoleucine; 4, Leucine; 5, 2-Aminobutyrate; 6, Valine; 7, Methylsuccinate; 8, Ethanol; 9, 3-Hydroxybutyrate; 10, Lactate; 11, Alanine; 12, Cadaverine; 13, Ornithine; 14, Acetate; 15, Glutamate; 16, Glutamine; 17, Glutathione; 18, Succinate; 19, Creatine; 20, Aspartate; 21, Malate; 22, Dimethylamine; 23, Choline; 24, O-Phosphocholine; 25, Carnitine; 26, Glucose; 27, Betaine; 28, Methanol; 29, Glycine; 30, Glycerol; 31, Ethylene glycol; 32, UDP-glucose; 33, UDP-glucuronate; 34, UDP-galactose; 35, Uridine; 36, NAD+; 37, Fumarate; 38, Tyrosine; 39, 3-Indoxylsulfate; 40, 3-Phenyllactate; 41, Nicotinurate; 42, NADP+; 43, Adenosine; 44, Inosine; 45, Formate; 46, ADP/AMP.

**Figure 7:**
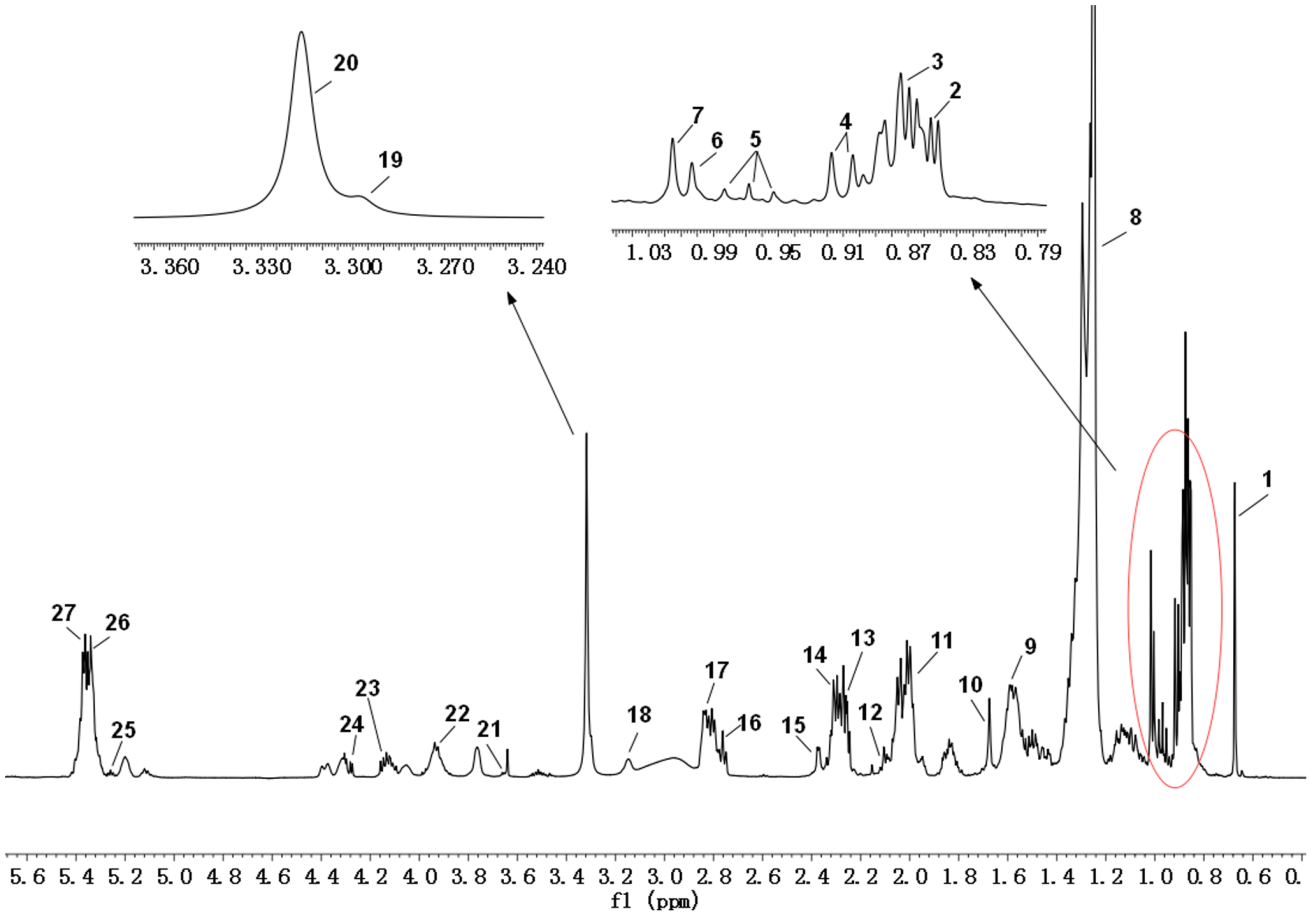
500 MHz ^1^H NMR spectra of lipophilic liver extract. 1, Total Cholesterol (C_18_ **H**_3_); 2, Total Cholesterol(C_26_ **H**_3_, C_27_ **H**_3_ **)**; 3, Fatty acid residues (ω-C**H**_3_); 4, Total Cholesterol (C_21_ **H**_3_); 5, Fatty acid residues (ω-C**H**_3_ of DHA+EPA+linolenic); 6, Free Cholesterol (C_19_ **H**_3_); 7, Esterified Cholesterol (C_19_ **H**_3_); 8, Fatty acid residues ((C**H**_2-_)_n_); 9, Fatty acid residues (COCH_2_-C**H**_2_); 10, Fatty acid residues (−C**H**_2_ of ARA+EPA); 11, Fatty acid residues (C**H**_2_-CH = ); 12, Fatty acid residues (*γ* C**H**_2_ of ARA+EPA); 13, Monoglycerides(FA, RH -C**H_2_**-CO-O-C_2_); 14, Fatty acid residues (−CO-C**H**_2_); 15, Fatty acid residues (α and β C**H**_2_ of DHA); 16, Fatty acid residues (−CH = CH-C**H**_2_-CH = CH-of linoleic acid); 17, FA, PUFA (CH = CH-C**H**_2_-(CH = CH-CH2)_n_, n>1); 18, Phosphatidylethanolamine **(**−C**H**_2_-NH_2_); 19, Sphingomyelin (−CH_2_-N-(C**H**_3_)_3_); 20, Phosphatidylcholine (−CH_2_-N-(C**H**_3_)_3_); 21, Cholesterol (C_3_ **H**); 22, Total phospholipids (Glycerol (C_3_ **H_2_**)); 23, Triglycerides (C_1_ **H** and C_3_ **H** of glycerol); 24, Triglycerides (C_1_ **H** and C_3_ **H** of glycerol); 25, Triglycerides(C_2_ **H** of glycerol); 26, Fatty acid residues (−C**H** = C**H**-); 27, Cholesterol (C_6_ **H**). ARA, Arachidonic acid; EPA, Eicosapentaenoic acid; DHA, Docosahexaenoic acid.

**Figure 8:**
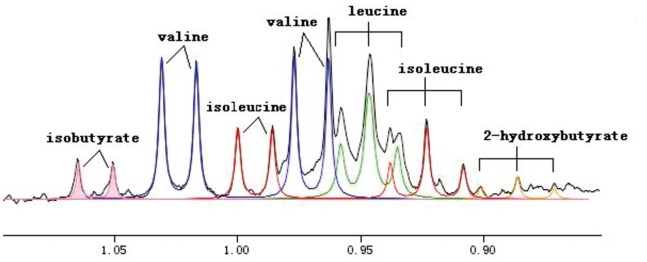
Representative quantifications and corresponding spectral regions of some plasma metabolites together with the mathematical fit with Chenomx. Assignments: 2-hydroxybutyrate (yellow); isoleucine (red); leucine (green); valine (blue); isobutyrate (pink).

Analysis of the NMR spectra of hamster plasma, urine and liver extract specimens revealed the wide variety of metabolite resonances present in the spectra. Representative ^1^H-NMR spectra of plasma, urine and liver extract samples are shown in Figures 4–7. The resonances were assigned to specific metabolites (Tables S1–S3) according to the literature data [7], [19], [20], the Chenomx metabolite database and extensive 2D NMR analysis, including ^1^H-^1^H COSY, ^1^H-^1^H TOCSY, ^1^H-^13^C HSQC and ^1^H-^13^C HMBC spectroscopy. A number of different types of metabolites, including amino acids, saccharides (glucose and galactose), compounds related to energy metabolism (pyruvate, succinate, citrate, lactate, creatine and creatinine), and other molecules (cholines, amines and amides) were identified. The quantified metabolites from filtered plasma, urine and aqueous liver extract, as well as corresponding resonances used for quantification, are listed in Tables S4–S6. Figure 8 depicts the quantifications and corresponding spectral regions of some of the most informative metabolites of plasma together with the mathematical fit with Chenomx NMR Suite.

#### 1H-CPMG-NMR spectra of plasma samples

**Figure 9:**
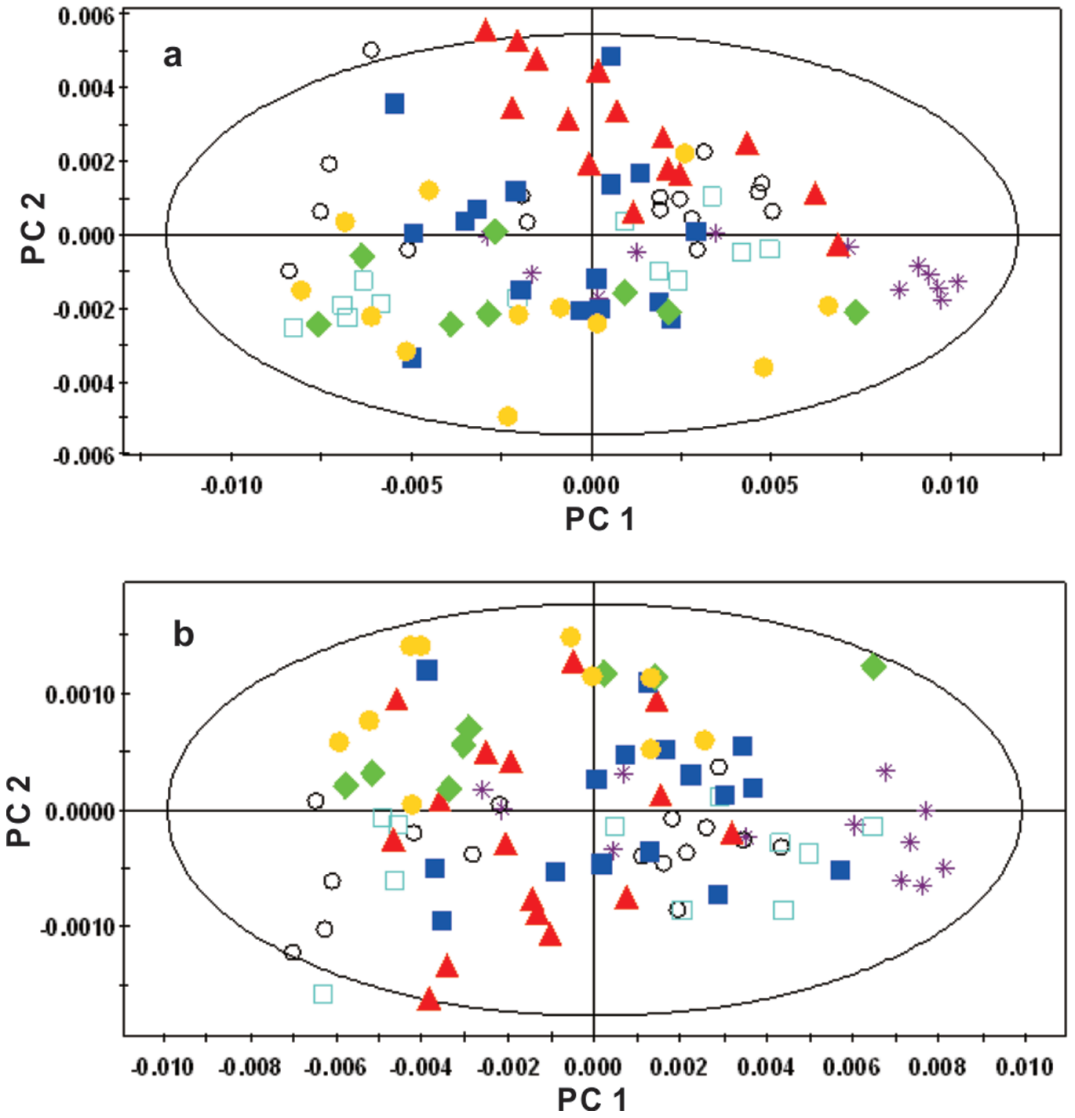
Trajectory derived from PCA of ^1^H-NMR spectra of hamster plasma from control groups at different time points (▴, week 0; ▪, week 3; ♦, week 9; •, week 15; *, week 26; ○, week 35; □, week 42). (a) Trajectory of ^1^H-CPMG-NMR spectra at different time points (two PCs, R^2^X = 0.896; Q^2^ = 0.391). (b) Trajectory of ^1^H- LEDBP-NMR spectra at different time points (two PCs, R^2^X = 0.968; Q^2^ = 0.835).

**Figure 10:**
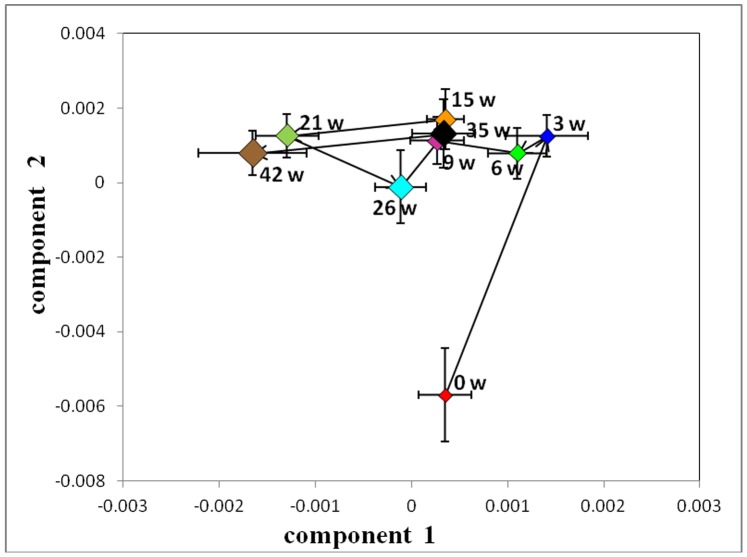
Trajectory derived from PCA of ^1^H-CPMG-NMR spectra of hamster plasma normalized on the sum of the spectrum mapping the time-related trajectory of metabotypes at weeks 0, 3, 6, 9, 15, 21, 26, 35 and 42.

To establish the time course of metabolic changes that were induced in hamsters by a HFHC diet, a PCA model was constructed for the ^1^H-NMR data obtained from the control and HFHC groups at each time point. The control group showed no significant differences over the entire period (Fig. 9). The averaged PCA scores of the HFHC group (n = 20) were calculated for the first two PCs. The CPMG trajectories (Fig. 10) showed that the HFHC group moved away from the 0-week position to the 3-week position along the PC2 axis onward, with decreased levels of citrate, succinate, lysine and proline. From the 3^rd^ week, the HFHC group moved away along the PC1 axis with a maximum shift reached at the end of the 42^nd^ week. During this time period, the metabolites that were significantly increased included betaine, glucose, ethanol and 2-hydroxybutyrate.

#### 1H- BPP-LED-NMR spectra of plasma samples

**Figure 11:**
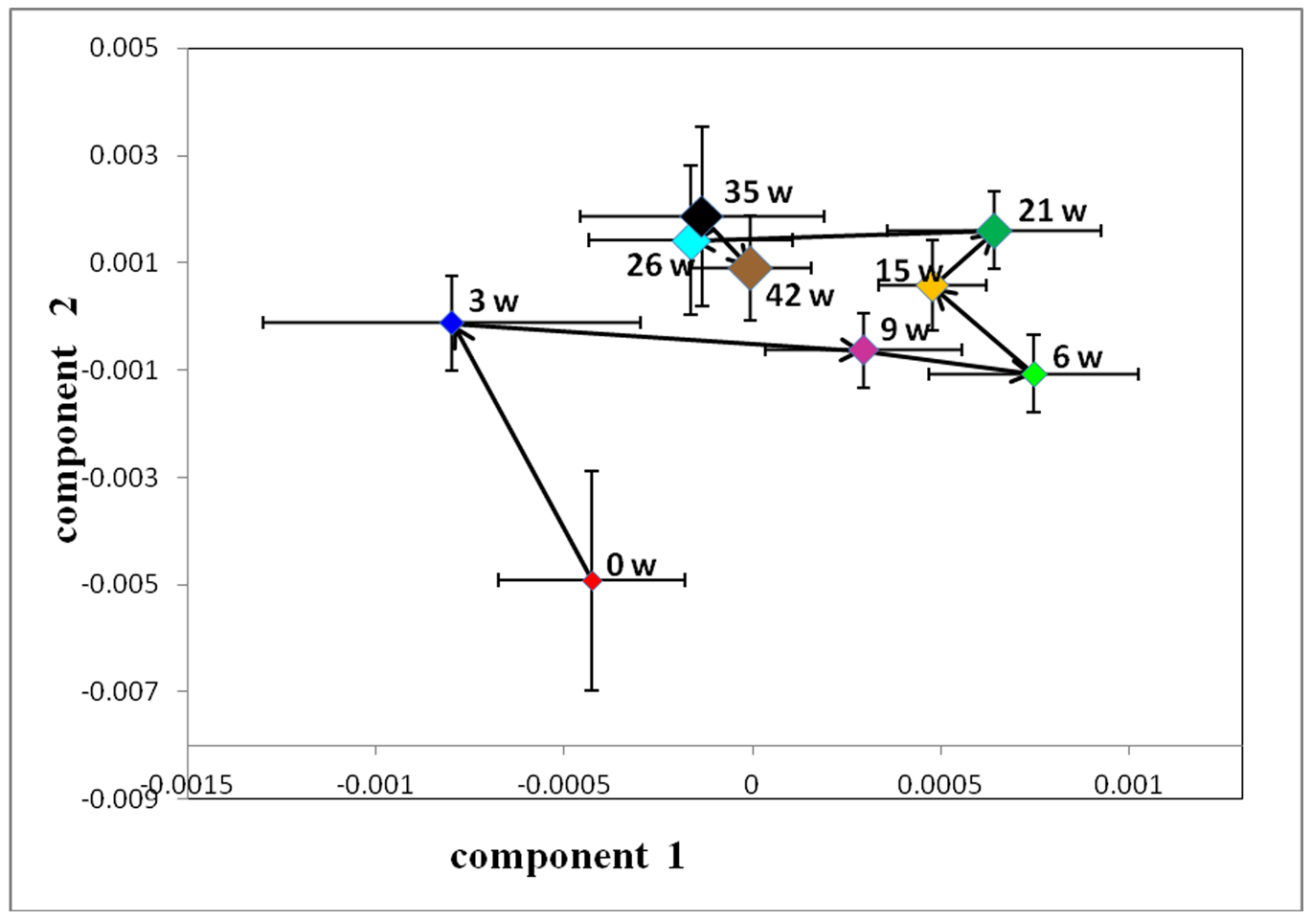
Trajectory derived from PCA of ^1^H-LEDBP-NMR spectra of hamster plasma normalized on the sum of the spectrum mapping the time-related trajectory of metabotypes at weeks 0, 3, 6, 9, 15, 21, 26, 35 and 42.

The LEDBP trajectories of HFHC group were similar to those of CPMG (Fig. 11). At the 3^rd^ week, the levels of low-density lipoprotein (LDL)/very-low-density lipoprotein (VLDL) and some lipids were significantly increased, while the levels of HDL, phosphatidylcholine (PtdCho) and unsaturated lipids were decreased, according to the loading plot. From the 3^rd^ week to the final time point, the levels of LDL/VLDL and N-acetyl glycoproteins progressively increased.

These results suggested that metabolic changes were closely correlated to the ongoing HCHF diet. To investigate the changes in detail, a pair wise comparative OPLS-DA model was calculated for the control and HFHC group at each time point, and a clear separation was obtained between the two groups at each time point. According to the loading plots, the metabolic perturbations taking place in the hyperlipidemic hamsters were mainly characterized by decreased levels of citrate, HDL, glycerol, phosphatidylcholine (PtdCho) and unsaturated lipids, as well as increased levels of LDL/VLDL, N-acetyl glycoproteins, betaine, and some lipids. These results are indicative of significant differences in the metabotypes of hamster plasma samples at various stages of hyperlipidemia, and some of our observations (e.g., changes in levels of LDL/VLDL) were also consistent with the findings of conventional blood biochemical analyses (Table 1).

#### Quantitative analysis of plasma metabolites

**Figure 12:**
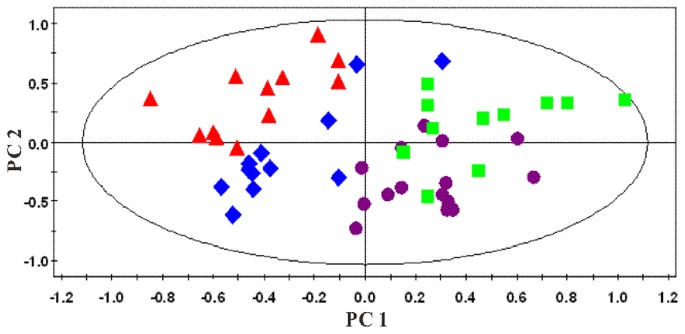
PCA score plots of plasma based on targeted profiling of 40 measured metabolites (▴, week 0; ♦, week 4; ▪, week 24; and •, week 42. R^2^X = 0.355, Q^2^ = 0.346).

**Figure 13:**
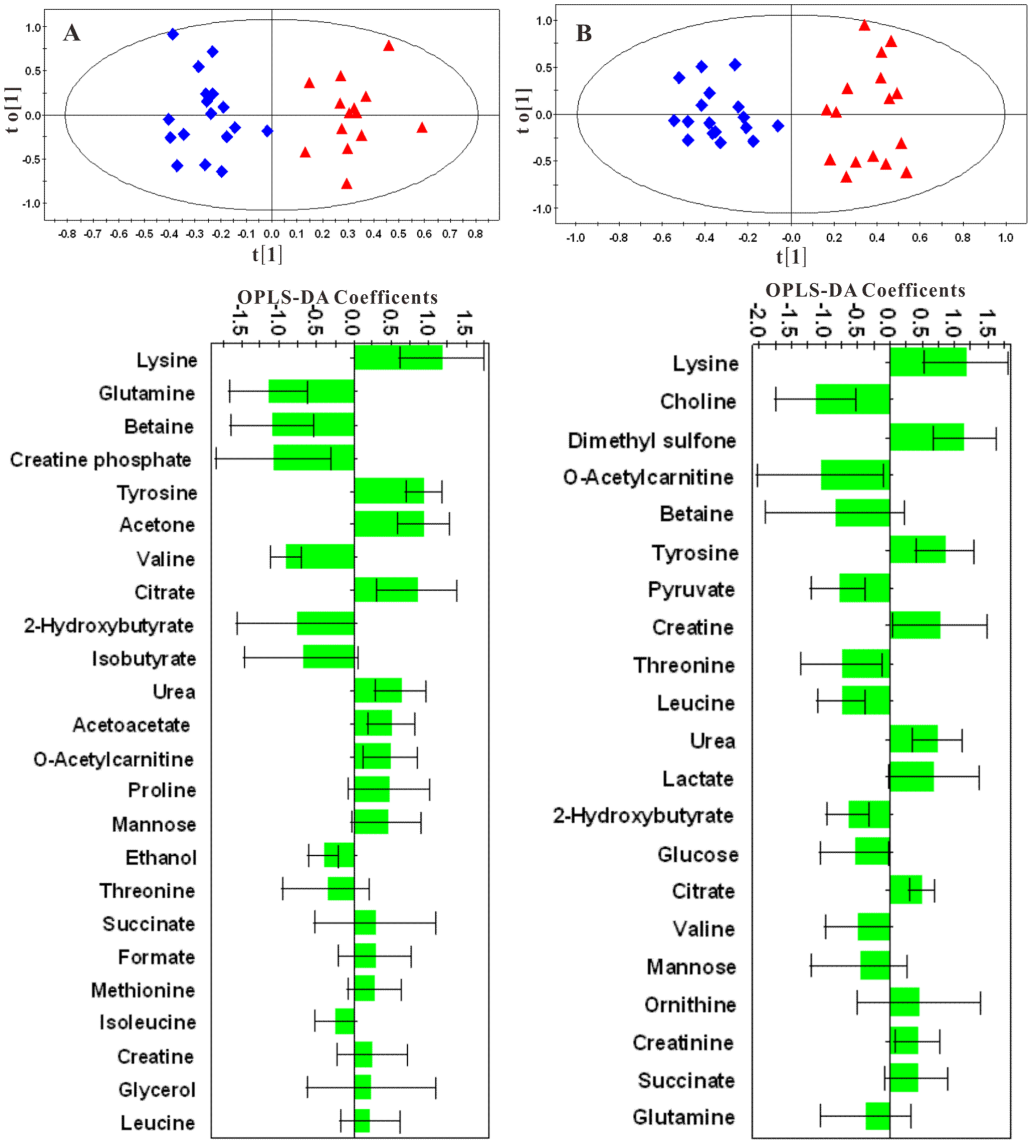
OPLS-DA scores (upper panel) and correlation coefficient plots (lower panel) derived from NMR data for hamster plasma samples at week 24 (A) and week 42 (B) (▴, control; ♦, HFHC). Positive bars (± SEM) of correlation coefficient plots denote metabolites significantly higher in control group, whereas negative bars (± SEM) denote metabolites significantly increased in the HFHC group.

An overview of the global changes occurring in plasma metabolites during the 42 weeks of dietary challenge was constructed by PCA using all the plasma data collected, resulting in a time-dependent trajectory of metabolite changes on the score plot (Fig. 12). Changes in plasma metabolites for the HFHC and the control hamsters at different time points were evaluated by OPLS-DA comparisons of the ^1^H NMR profiles of the control and group and the HFHC group at the same time point. For illustrative purpose, cross validated scores plot and corresponding coefficient plots of different time points were displayed in Figure 13. The pair wise comparative OPLS-DA of the NMR data showed significant intergroup metabolomic differences with good model quality indicated by the R^2^X and Q^2^ values (Table 2). The results of the permutation tests further suggested that the models constructed from the spectral data at week 4, week 24 and week 42 were valid.

**Table 2:** Correlation Coefficients from OPLS-DA of hamster plasma at week 4, week 24 and week 42^a^.

	Week 4	Week 24	Week 42
	R^2^X = 0.296	R^2^X = 0.449	R^2^X = 0.529
Metabolites	Q^2^ = 0.408	Q^2^ = 0.688	Q^2^ = 0.75
2-Hydroxybutyrate	−1.18	−0.77	−0.65
Betaine	−1.38	−1.09	−0.85
Choline	−0.69	−0.2	−1.14
Citrate	0.06	0.85	0.49
Creatine	0.02	0.24	0.77
Ethanol	0.01	−0.4	0.01
Glucose	−0.04	0.04	−0.55
Inosine	0.31	−0.12	0.19
Isobutyrate	0.49	−0.7	0.05
Lysine	0.43	1.18	1.16
Methionine	0.55	0.27	0.03
Pyruvate	0.19	−0.14	−0.79
Tyrosine	0.03	0.93	0.84
Urea	0.32	0.62	0.73

a A positive value indicates an increase in the control group, and a negative value indicates an increase in the HFHC group.

#### Quantitative analysis of urine samples

**Figure 14:**
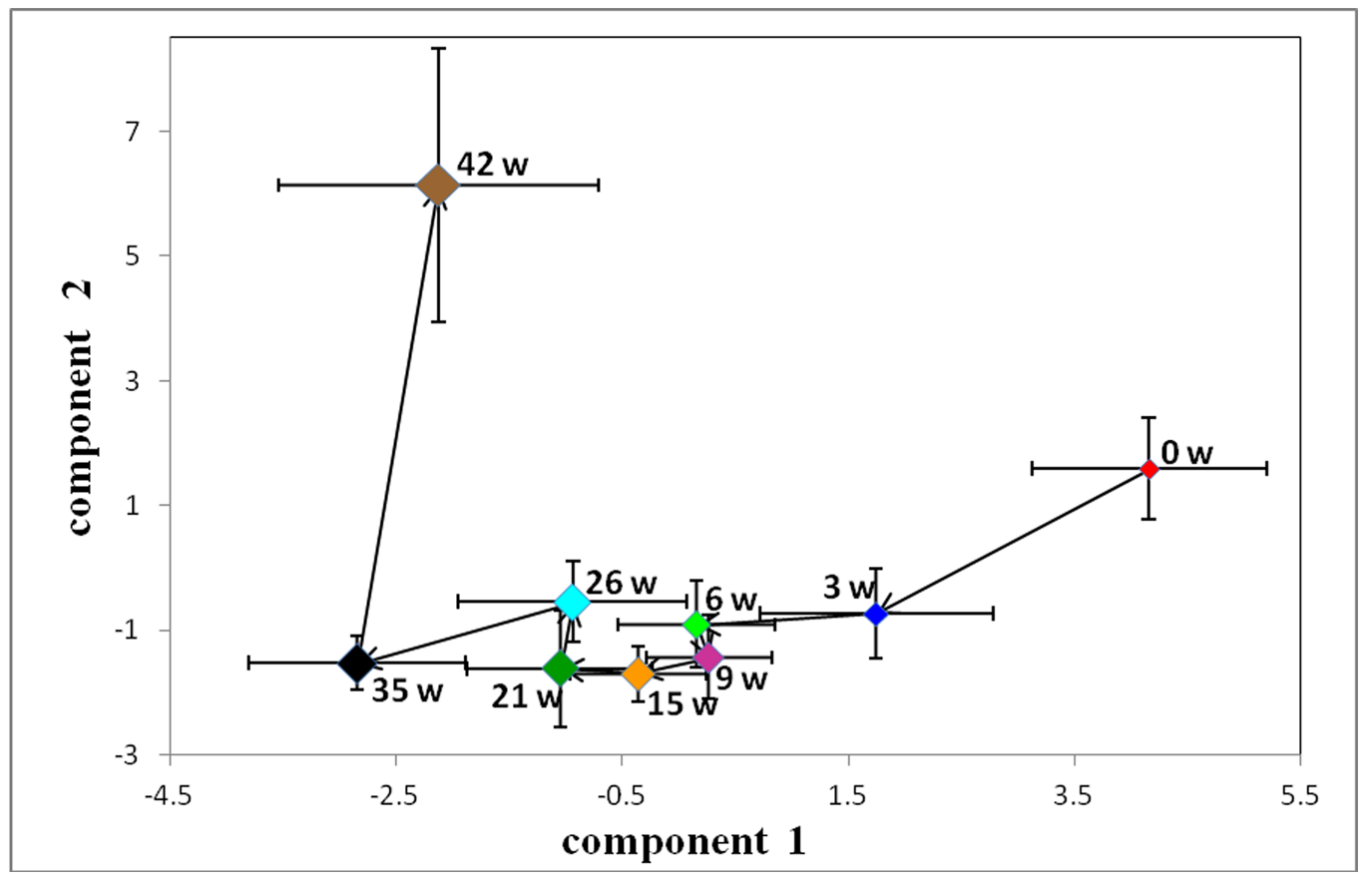
Trajectory derived from PCA of targeted profiling of 80 measured urine metabolites (normalized to the total concentration of all measured metabolites) revealed metabolic changes associated with dietary treatment.

**Figure 15:**
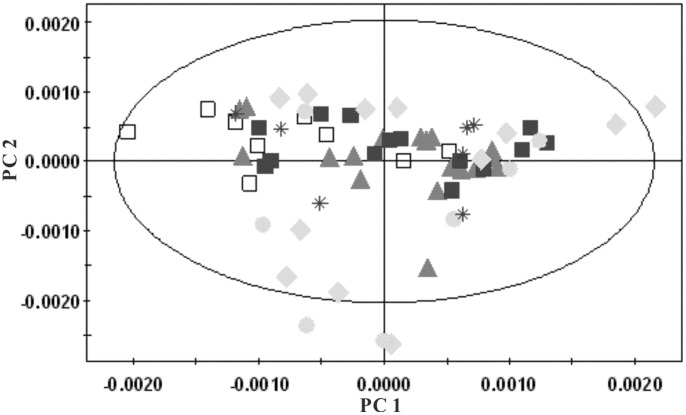
PCA score plots of ^1^H-NMR spectra of hamster urine from control groups at different time points(▴, week 0; ▪, week 6; ♦, week 15; •, week 26; *, week 35; □, week 42).

**Figure 16:**
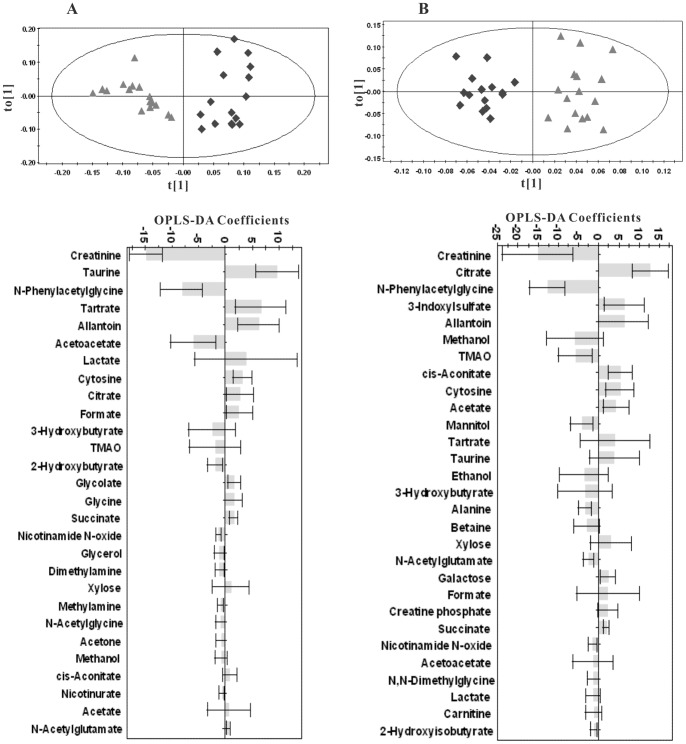
OPLS-DA scores (upper) and correlation coefficient plots (lower) derived from NMR data for hamster urine samples at week 15 (A) and week 42 (B) (▴, control; ♦, HFHC). Positive bars (± SEM) of correlation coefficient plots denote metabolites significantly higher in control group, whereas negative bars (± SEM) denote illustrate metabolites.

**Figure 17:**
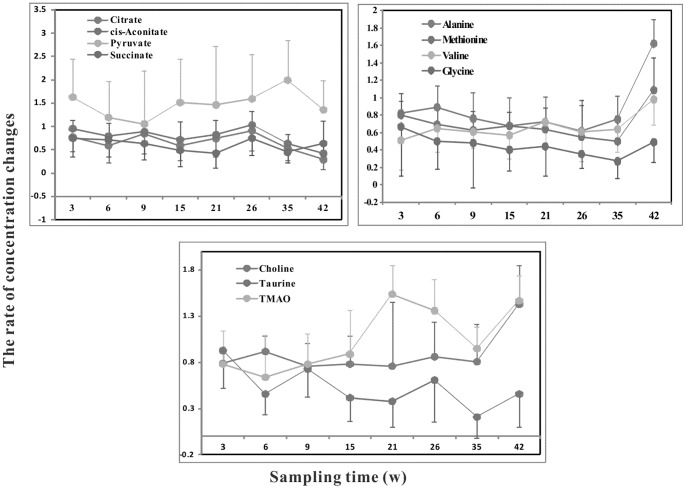
Temporal changes in individual metabolite levels in the urine samples of HFHC animals at weeks 3, 6, 9, 15, 21, 26, 35 and 42. The Y axis values were calculated based on the measured metabolite concentrations in the untreated control group with the formula Cm/Cc, where Cm represents the respective metabolite concentration in the HFHC group and Cc represents that in the control group. The error bars represent the standard deviation of the mean.

Similar to the plasma samples, an overview of the global changes occurring in urine metabolites during 42 weeks of dietary challenge was constructed by PCA using all the urine data collected. The trajectory of the HFHC group (Fig. 14) showed time-dependent changes that were similar to the changes observed in the plasma samples of the HFHC group. In contrast, the control group showed no significant differences over the entire period (Fig. 15). From week 0 to 35, the trajectory was mainly changed along the PC1 axis, with a maximum shift reached at the end of the 35^th^ week. From the 35^th^ week to 42^nd^ week, the direction of changes in the metabolic trajectory switched to the PC2 axis. According to the loading plot, the main changes in metabolites from week 0 to the 35^th^ week were increased levels of 3-hydroxybutyrate, acetoacetate, acetone, isobutyrate, pyruvate and nicotinurate and decreased levels of taurine, citrate, succinate, cis-aconitate, acetate, tyrosine, and cytosine. At the 42^nd^ week, the levels of choline, betaine, alanine, nicotinamide N-oxide, 2-hydroxyisobutyrate, N-dimethylglycine and N-acetylglutamate were significantly increased compared to the levels measured at the 35^th^ week. The diet-related metabolic effects were evaluated via OPLS-DA comparisons of the ^1^H-NMR profiles of the control and HFHC group samples collected at the same time point. For illustrative purposes, Figure 16 depicts the cross-validated score plot and the corresponding coefficient plots derived from the data of selective time points. The pair wise comparative OPLS-DA of the NMR data showed significant intergroup metabolomic differences, with good model quality indicated by the R^2^X and Q^2^ values. The results of the permutation tests further suggested that the models constructed from the spectral data at the 3^rd^, 6^th^, 9^th^, 15^th^, 21^st^, 26^th^, 35^th^ and 42^nd^ weeks were valid. The important metabolites that were significantly altered at different time points as a result of the dietary treatment are summarized in Table 3. Figure 17 illustrates the temporal changes occurring in the main metabolites that were significantly affected by the HFHC diet. The three parts of Figure 17 respectively represent the temporal changes of metabolites related to glucose, amino acid, and gut microbiota metabolism. The most remarkable change observed was the long-lasting increase in the levels of 3-hydroxybutyrate, acetoacetate, acetone, creatinine, isobutyrate, 2-hydroxybutyrate, 2-hydroxyisovalerate, nicotinamide N-oxide, Pyruvate, and N,N-dimethylglycine. Meanwhile, the levels of hypoxanthine, acetate, cytosine, 3-indoxylsulfate, taurine, tartrate, citrate, creatine, dimethylamine, and cis-aconitate were decreased. The levels of trimethylamine N-oxide, hippurate, alanine, and N-acetylglutamate were decreased at the early time points, and their levels increased at later time points.

**Table 3:** Correlation coefficients from OPLS-DA of hamster urine at weeks 3, 6, 9, 15, 21, 35 and 42^a^.

Metabolites	Week 3	Week 6	Week 9	Week 15	Week 21	Week 35	Week 42
	R^2^X = 0.897	R^2^X = 0.891	R^2^X = 0.923	R^2^X = 0.944	R^2^X = 0.832	R^2^X = 0.874	R^2^X = 0.858
	Q^2^ = 0.576	Q^2^ = 0.733	Q^2^ = 0.496	Q^2^ = 0.789	Q^2^ = 0.651	Q^2^ = 0.826	Q^2^ = 0.800
2-Hydroxybutyrate	−3.19	−0.81	−4.45	−1.96	−0.59	−0.99	−0.56
3-Hydroxybutyrate	−13.85	−4.63	−7.87	−2.48	−3.97	−4.12	−3.44
3-Indoxylsulfate	3.58	0.90	4.83	0.53	0.70	0.68	6.24
Acetate	2.59	3.51	2.70	0.65	1.60	1.76	4.32
Acetoacetate	−10.70	−4.94	−8.01	−6.02	−5.42	−4.89	−1.48
Acetone	−5.73	−0.74	−1.61	−0.94	−0.62	−0.70	−0.49
Alanine	2.26	0.02	1.60	0.46	0.25	−0.01	−3.42
Choline	1.16	0.07	0.95	0.19	0.06	−0.08	−0.88
Citrate	8.57	3.47	1.10	2.64	0.53	1.38	12.74
Creatinine	−15.37	−10.14	−22.40	−14.88	−2.82	−1.16	−15.11
Creatine	−0.33	−0.03	1.62	0.30	0.42	0.21	0.46
Cytosine	7.75	2.68	9.21	3.10	1.91	1.19	5.32
Dimethylamine	2.83	0.04	0.87	−1.12	−0.11	0.02	0.59
Galactose	2.49	0.42	−6.43	0.53	0.63	0.25	2.31
Hippurate	2.87	−0.20	1.72	−0.06	−0.64	−0.65	−0.65
Hypoxanthine	1.10	0.15	0.94	0.37	0.20	0.21	0.45
Isobutyrate	0.56	−0.03	0.01	−0.03	0.09	−0.01	0.05
Methionine	0.68	0.04	0.82	0.16	0.13	0.14	0.15
N,N-Dimethylglycine	−1.19	−0.49	−1.25	−0.32	0.01	−0.16	−1.38
N-Acetylglutamate	1.89	−0.09	0.92	0.56	0.35	−0.16	−2.56
Nicotinamide N-oxide	−1.02	−0.70	−2.79	−1.40	−0.45	−0.50	−1.53
Pyruvate	−1.30	−0.03	0.95	−0.22	−0.08	−0.35	−0.69
Succinate	2.89	0.80	2.57	1.47	0.88	0.38	1.90
Tartrate	14.02	−1.36	11.45	6.57	3.27	1.06	4.03
Taurine	−11.78	11.60	−6.32	9.75	9.98	9.93	3.83
Trimethylamine N-oxide	3.89	1.32	−0.54	−1.99	−2.82	−0.38	−5.79
cis-Aconitate	1.16	0.33	0.03	0.82	0.16	0.30	5.33

a A positive value indicates an increase in the control group, and a negative value indicates an increase in the HFHC group.

#### 1H-NMR spectra of liver extract

**Figure 18:**
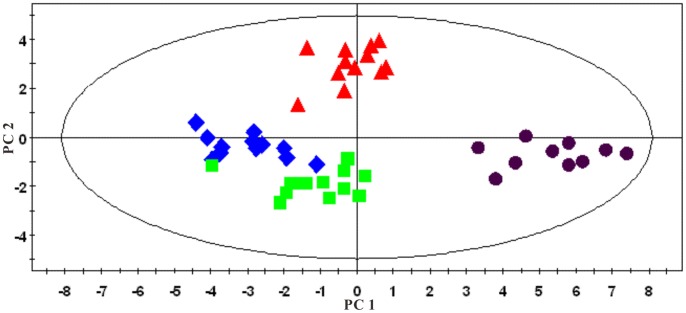
Multivariate data analyses of the ^1^H-NMR spectra of the water-soluble fraction of liver extracts at different time points: ▴, week 0; ♦, week 4; ▪, week 24; and •, week 42. R^2^X = 0.582; Q^2^ = 0.494.

**Figure 19:**
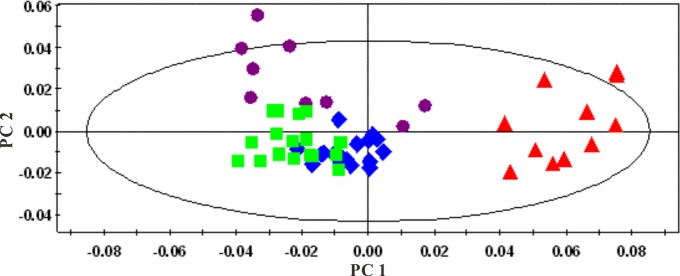
PCA plots derived from ^1^H -NMR spectra of the organic phase of liver extracts at different time points: ▴, week 0; ♦, week 4; ▪, week 24; and •, week 42; R^2^X = 0.753; Q^2^ = 0.703.

PCA of both water-soluble extract and lipophilic extract were conducted. A series of characteristic time-dependent changes was revealed on the spectra results, resulting in a time-dependent trajectory appearing on the plot (Fig. 18 and 19). Changes in the liver metabolome of the HFHC and control hamsters at different time points were established using the PLS-DA approach, comparing the ^1^H NMR profiles of control and HFHC groups to assess metabolite concentrations. Clear separation at each time point was achieved between samples obtained from the control and HFHC group, as evidenced by the consistently high Q^2^Y values for all models. The important metabolites responsible for the separation of the water-soluble fraction of the liver extract of the HFHC group from the control group at different time points are summarized in Table 4. Analysis of the loading plots of liver lipophilic extract, the HFHC groups showed significantly higher hepatic levels of triglycerides, free and esterified cholesterol and oleic acid, but decreased phosphatidylcholine, polyunsaturated fatty acids (PUFA) (ω-3 fatty acyls, docosahexanoic (22∶6n-3; DHA) acids, arachidonic (20∶4n-6; ARA)+eicosapentaenoic (20∶5n-3; EPA) acids), mono-unsaturated fatty acids (MUFA) and [PUFA/MUFA] ratios.

**Table 4:** Correlation coefficients from OPLS-DA of metabolites of hamster liver water-soluble extract at weeks 4, 24 and 42^a^.

Metabolites	Week 4	Week 24	Week 42
	R^2^X = 0.95	R^2^X = 0.952	R^2^X = 0.912
	Q^2^ = 0.725	Q^2^ = 0.474	Q^2^ = 0.698
2-Aminobutyrate	−3.62	−6.87	−8.85
2-Hydroxybutyrate	−1.18	−2.45	−1.52
3-Hydroxybutyrate	−29.01	85.17	23.88
Acetate	1.32	10.29	4.70
Adenine	3.92	5.96	2.34
Adenosine	7.12	17.71	5.41
Alanine	119.11	59.61	−26.32
Aspartate	−12.09	−35.15	−89.60
Betaine	−187.71	−87.65	−207.07
Cadaverine	7.07	19.95	10.28
Choline	3.43	47.07	37.77
Creatine	1.34	7.76	5.69
Ethanol	41.96	−24.12	−83.36
Fumarate	−1.83	−26.96	−21.85
Glucose	−8.19	−24.10	−36.17
Glutamate	−20.14	−13.14	−49.58
Glutamine	−251.17	−297.37	−130.81
Glutathione	2.69	−13.84	32.87
Glycerol	−3.27	24.60	39.05
Lactate	162.81	−64.90	92.25
Methanol	−13.27	−64.24	9.97
N,N-Dimethylglycine	1.43	4.16	1.58
NAD+	5.67	−6.39	−8.70
NADP+	−1.69	−5.62	−0.97
Nicotinurate	5.20	15.12	9.84
O-phosphocholine	−3.88	18.96	34.58
Ornithine	14.16	12.04	9.06
Succinate	6.91	6.97	30.86
Taurine	19.49	86.38	131.95

a A positive value indicates an increase in the control group, and a negative value indicates an increase in the HFHC group.

## Discussion

This is the first study examining the feasibility of using a combination of non-quantitative and quantitative metabonomics to determine the severity of diet-induced hyperlipidaemia. The hyperlipidemic hamster model used in our study has been previously found to be appropriate for the modeling of hyperlipidaemia by dietary challenge [7], [21]. The combination of non-quantitative and quantitative metabonomics can comprehensively measure the multiparametric metabolic responses of biological systems. In our study, plasma, urine and liver samples were collected at multiple time points and a set of metabolites were assayed to identify differences in concentration. The results revealed that endogenous metabolites in hamster plasma, urine and liver specimens all showed time-dependent changes at different stages of hyperlipidaemia, indicating the progressive development of hyperlipidemia. In addition, our approach revealed hyperlipidaemia-associated changes in lipid metabolism, inflammation, oxidative stress and gut microbiota metabolites, thereby highlighting the multifaceted nature of hyperlipidaemia. Thus, our results indicate that the combination of non-quantitative and quantitative metabonomics provides a holistic approach for determining the response of an intact system to chemical and physical perturbations, leading to a broader view of the metabolic network.

### Metabolites Related to Lipid Metabolism

Lipid metabolism disorders play a pivotal pathogenic role in the initiation and development of hyperlipidemia, and are believed to trigger cardiovascular arterial disease. Besides the disturbed levels of TG and total choline-containing metabolites (t-CHO), which were previously revealed by the traditional biochemical assays, we also observed other remarkable abnormalities in the lipid metabolism of hyperlipidemic hamsters, including elevated plasma levels of LDL/VLDL from week 1 through 42 and decreased plasma levels of HDL and unsaturated lipids. These results are consistent with the results obtained using traditional biochemical assays. In addition, a significant decreased level of phosphatidylcholine (PtdCho) in the plasma and liver of HFHC groups is consistent with a decrease in the HDL level, as PtdCho is the most predominant lipid in the HDL fraction [22]–[25]. Besides, since VLDL synthesis requires phospholipids, particularly PtdCho, an insufficiency of PtdCho or its precursors can lead to the decreased secretion of triglycerides from the liver, giving rise to fatty liver. Moreover, a decreased PUFA/MUFA ratio is observed at an early stage, probably indicating excessive lipid peroxidation and oxidative stress. The significant depletion of hepatic PUFA indicates a reduction in triglyceride release from the liver with a consequent increase in triglyceride synthesis that may contribute significantly to the development of triglyceride accumulation in hepatocytes [20]. This interpretation is consistent with the result of Oil Red O staining and ultrasonographic imaging. Increased fatty acid oxidation requires nicotinamide-adenine dinucleotide (NAD+) for normal mitochondrial acid fatty oxidation [26]. Therefore, higher levels of nicotinamide N-oxide, a precursor for NAD+ in animals that can be converted to NAD+ by xanthine oxidase in the liver [27], was observed in the hyperlipidemia model hamsters.

### Metabolites Related to Energy Homeostasis

While the level of glucose did not undergo any obvious changes during the first 24 weeks of dietary challenge, a significant increase in plasma glucose was observed in the 42^nd^ week, along with a marked increase in pyruvate levels. The increased glucose may be the result of slowed glycolysis. Pyruvate, a key metabolite important to both glycolysis and the tricarboxylic acid (TCA) cycle, can be converted into acetyl-CoA by decarboxylation and enter the TCA cycle under aerobic conditions. However, hyperlipidemia can lead to a reduction of dissolved oxygen in the media and plasma, slowing the conversion of pyruvate to acetyl-CoA and resulting in a high level of pyruvate. We found that levels of Kreb’s cycle intermediates, including plasma citrate and succinate, as well as urine citrate, succinate and cis-aconitate, were all decreased throughout the entire experiment, with a dramatic reduction occurring at the 42^nd^ week. These results demonstrate that the aberrant changes in glycolysis and the TCA cycle occurred at various stages of hyperlipidaemia. The reduced flux through the TCA cycle and increased rate of fatty acid oxidation may indicate that vascular cells respond to a high-fat/high-cholesterol diet by subjoining the metabolism of lipids. Increased oxidation of fatty acids may produce short- or medium-chain fatty acids (the primary oxidative substrates), while progressively slowing down glucose metabolism (the main energy source of the vasculature), and the energy consumption is switched to lipid oxidation [28]. In addition, decreases in the level of plasma creatine were also seen from the 4^th^ week through the 42^nd^ week, and urine creatine began to decrease at the 9^th^ week of treatment. The creatine-phosphocreatine system is crucial for transportation of energy produced by the mitochondria [29]. The decreased levels of creatine we observed are therefore consistent with a disruption of energy homeostasis. Interestingly, reduced serum creatine levels in humans and mouse models has recently been reported in a metabolomic profiling studies of steatosis [30], [31] and NAFLD progression [32]. Besides, creatine and creatinine can reflect the injury of both liver and kidney, so their changes may be a sign of hepatic and renal insufficiency with long-term exposure to the HFHC diet [33].

### Amino Acids and Related Metabolites

Hyperlipidaemia may also affect amino acid metabolism, leading to changes in the levels of many amino acids, including leucine, methionine, lysine, alanine, glycine and valine. Methionine is an intermediate in a transmethylation reaction that uses S-adenosyl methionine (SAM) as a methyl donor to produce homocysteine [34]. By acting as a methyl donor during the remethylation of homocysteine, betaine converts homocysteine into methionine and helps maintain the appropriate level of SAM. The blood and urine levels of methionine decreased in the fourth week, while betaine content was significantly increased in the early phase of hyperlipidemia (week 4) in the plasma and liver. Betaine was discovered to be the most important variable in predicting hyperlipidemia, and could be considered an early biomarker of coronary heart disease. The observed betaine and methionine changes suggest that the methionine/homocysteine cycle in these samples may be enhanced, leading to elevated homocysteine levels, which is considered to be a risk factor for cardiovascular disease. It has been found that high homocysteine levels are associated with hyperlipidemia, and hypomethylation caused by high homocysteine levels is the main reason for the enhanced uptake and synthesis of cholesterol and triglycerides by the liver, which eventually leads to the accumulation of fat in tissues [35]. These results are therefore in agreement with a connection between hyperlipidemia and hyperhomocysteinemia [36]. The concentration of plasma lysine began to decrease at the 4^th^ week, and this trend continued until the 42^nd^ week, which was consistent with previous reports [7]. The lysines in albumin- and apoB100-containing particles can generate glycosylation end products or can be further oxidized [37], [38], thus the lower free lysine level might indicate the occurrence of oxidative stress in the pathogenesis of hyperlipidemia, which might aggravate fatty liver disease and fat accumulation. Glutamate and glutamine are both precursors for two particularly important antioxidants, glutathione [39] and taurine. Previous studies in many patients found that plasma glutamate and glutamine concentrations could either surge or fall [40], suggesting that they might be important amino acids during the pathogenesis of metabolic diseases. For example, low plasma glutamate concentrations and low glutamate/glutamine ratios are used to predict the outcome of patients who have septic shock with acute liver dysfunction [41]. In our study, the significant increase in glutamate/glutamine levels in the livers of HFHC-fed hamsters might be the manifestation of abnormal liver function.

### Metabolites Related to Gut Microbiota

In urine, the levels of choline, trimethylamine-N-oxide (TMAO), hippurate, taurine and dimethylamine were all decreased at first 6 weeks and then increased during the experiment. TMAO is often derived from di- and trimethylamine generated during the metabolism of choline [42] or carnitine [43] by gut microbiota. This suggested the possibility of diet-mediated changes in the metabolic activity of gut microbiota during the pathogenesis of hyperlipidemia. Many recent metabolic studies have shown that gut microflora are closely associated with diet-induced obesity, and that consumption of a high-fat diet results in a decrease in total gut bacterial levels, leading to alterations of metabolites such as TMA and TMAO [44]–[46]. The alteration of hippurate levels also indicate disturbances in the gut microbiome of the hyperlipidemic hamsters [47]. Taurine, a sulfur-containing β-amino acid, is a major free intracellular amino acid found in many animal tissues. It is suggested to have a number of protective properties, including protection against hepatic damage [48]. In the liver, taurine is linked to the activity of the hepatic cholesterol-7R-hydroxylase (CYP7A1), a key enzyme in the process of cholesterol excretion and bile acid synthesis [49], [50]. As previously suggested, the decreased hepatic concentration of taurine in animals challenged with high-fat/high-cholesterol diets could be due to an increased excretion of taurine-conjugated bile acids caused by an excess of cholesterol in the liver [51].In addition, taurine, like glutathione, can also act as an antioxidant, and its change in abundance could indicate the occurrence of oxidative stress [52].

### Metabolites Related to Inflammation and other Metabolites

The levels of N-acetyl-glycoproteins that serve as inflammatory markers and acute-phase reactant proteins [53], such as serum amyloid A (apoSAA) and C-reactive protein (CRP) [54], were significantly increased during the early stages of hyperlipidemia (the 4^th^ week). Thus, increased levels of N-acetyl-glycoproteins are likely to indicate hyperlipidemia-induced inflammation in our study. The level of 3-indoxylsulfate, another oxidative stress-related metabolite, gradually diminished, which may suggest the presence of oxidative stress in the HFHC group. Nicotinamide N-oxide, a nicotinamide-associated metabolite closely related to reactive oxygen species (ROS) production, can also cause complications pathways relevant to obesity [55]. Therefore, the results suggested that disturbed carbohydrate metabolism might contribute to oxidative stress and inflammation in hamster models of hyperlipidemia. Moreover, the sharp reductions in nucleotide derivatives in urine during the entire course of treatment, such as cytosine, resulted from RNA degradation and cell turnover [56], and might reflect perturbations in nucleotide metabolism.

### Conclusions

**Figure 20:**
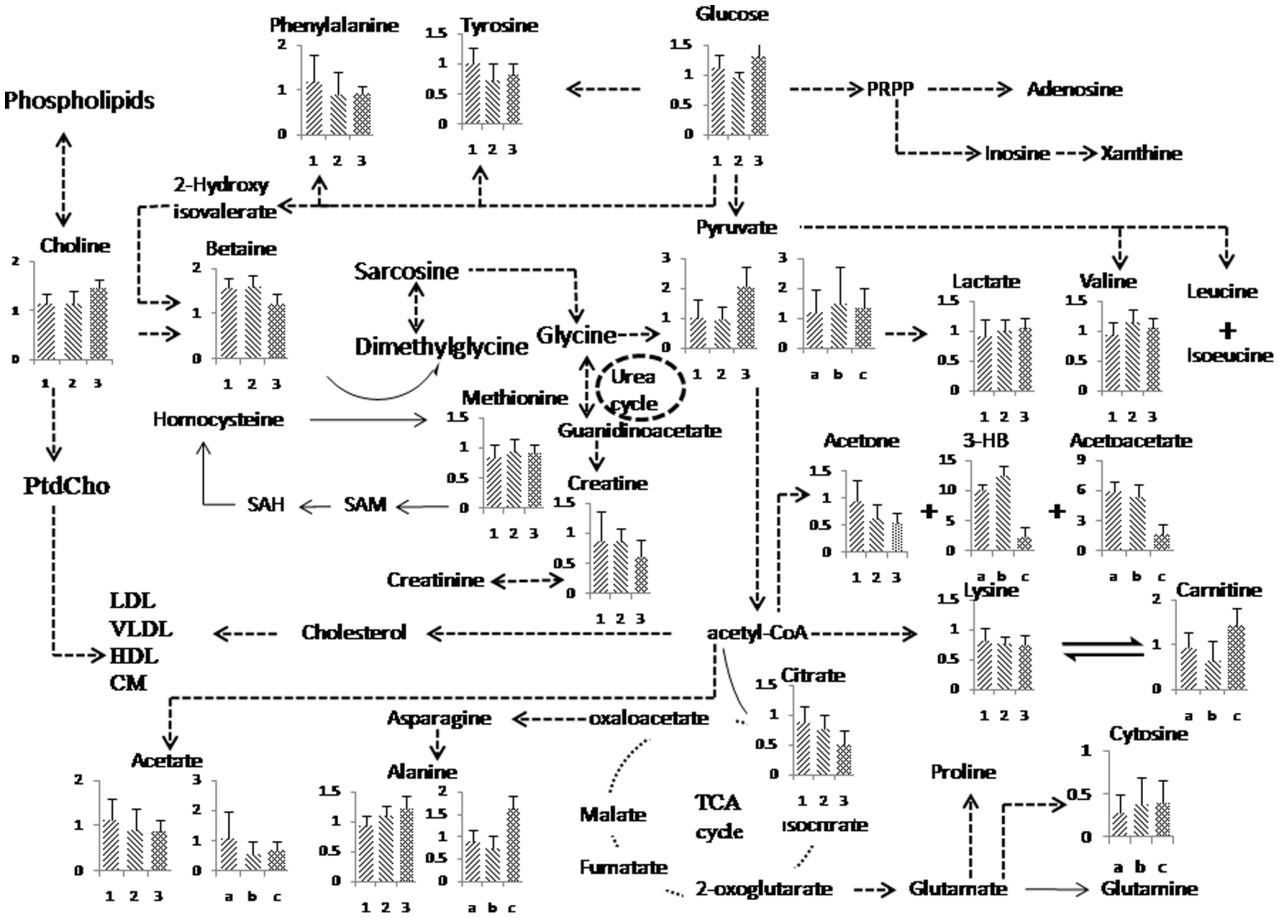
Schematic diagram of the hamster metabolic pathways and the relative levels of the major compounds within these pathways at various times in the development of hyperlipidaemia. 1, 2, and 3 stand for plasma samples taken at weeks 4, 21 and 35, respectively, while a, b and c stand for urine samples taken at weeks 6, 21 and 42, respectively.

The time-resolved analysis of the metabolomic responses of hamsters to a HCHF diet was carried out using non-quantitative and quantitative metabonomics. The dietary challenge was the obvious factor affecting the metabolic trajectory of the hamsters, and was mainly associated with a disturbed lipid and energy metabolism; however, it was also accompanied by altered amino acid and nucleotide metabolism, inflammation, oxidative stress and aberrant metabolic activity of gut microbiota (Fig. 20). At an early stage (the 3^rd^ week), the aberrant levels of TCA intermediates, fatty acid and ketone bodies indicated energy disorder, mainly caused by disturbed lipid metabolism. At a later stage, the changes in the levels of N-acetyl-glycoproteins, taurine, TMAO, choline, and dimethylamine may indicate inflammation, oxidative stress and the changes in gut microbiota metabolites, which are all associated with the progression of disease. At the last stage (42 weeks), the significant increase of glutamate and glutamine in liver extract may be a sign of liver dysfunction. The difference in NMR spectral profiles faithfully depicts the pathophysiological changes and metabolic disturbances observed at the different phases of the disease progression. This study has highlighted the benefits of a combined NMR-based metabolomics strategy to gain new insights into the nature of hyperlipidemia, and can be further used to develop multi-parameter approaches for better diagnosis and clinical management of this disease.

## Supporting Information

Table S1 **^1^H Chemical shift assignment of the metabolites in plasma from hamsters.**
(DOCX)

Table S2 **^1^H Chemical shift assignment of the metabolites in urine from hamsters.**
(DOCX)

Table S3 **^1^H Chemical shift assignment of the metabolites in liver from hamsters.**
(DOCX)

Table S4 **Quantitative metabolites in filtered plasma and corresponding^ 1^H Chemical shift assignment used for quantification.**
(DOCX)

Table S5 **Quantitative metabolites in urine and corresponding^ 1^H Chemical shift assignment used for quantification.**
(DOCX)

Table S6 **Quantitative metabolites in liver aqueous extracts and corresponding^ 1^H Chemical shift assignment used for quantification.**
(DOCX)
