# Supplementary material for: A 1H NMR-Based Metabonomic Investigation of Time-Related Metabolic Trajectories of the Plasma, Urine and Liver Extracts of Hyperlipidemic Hamsters
Source: PLoS One. 2013 Jun 26;8(6):e66786. doi: 10.1371/journal.pone.0066786 (PMC3694122; doi:10.1371/journal.pone.0066786)
Supplement: Table S5 — Quantitative metabolites in urine and corresponding 1H Chemical shift assignment used for quantification. (DOCX) [file pone.0066786.s005.docx]

| **Table S5.** Quantitative metabolites in urine and corresponding ^1^H Chemical shift assignment used for quantification | | |
| --- | --- | --- |
| **Metabolites** | **Moieties** | **δ^1^H(ppm)and multiplicity** |
| 2-Hydroxybutyrate | αCH | 3.99(dd,6.6,4.5Hz) |
|  | βCH_2_ | 1.64(m),1.73(m) |
|  | γCH_3_ | 0.89(t,7.5Hz) |
| 2-Hydroxyisobutyrate | CH_3_ | 1.36(s) |
| 2-Hydroxyisovalerate | αCH | 3.84(d) |
|  | βCH | 2.01(m) |
|  | γCH_3_ | 0.83(d) |
|  | γ'CH_3_ | 0.96(d) |
| 2-Hydroxyvalerate | αCH | 4.03(m) |
|  | βCH_2_ | 1.35(m) |
|  | γCH_2_ | 1.63(m) |
|  | δCH_3_ | 0.91(t) |
| 2-Oxoglutarate | βCH_2_ | 2.43(t,6.9Hz) |
|  | γCH_2_ | 2.99(t,6.8Hz) |
| 2-Oxoisocaproate | βCH | 2.61(d) |
|  | γCH | 2.08(m) |
|  | δCH_3_ | 0.92(d) |
| 3-Hydroxy-3-methylglutarate | CH_3_ | 1.31(s) |
| 3-Hydroxybutyrate | αCH | 4.15(m) |
|  | βCH_2_ | 2.34(m),2.38(m) |
|  | γCH_3_ | 1.19(d,6.3Hz) |
| 3-Hydroxyisovalerate | αCH | 3.84(d) |
|  | βCH | 2.01(m) |
|  | γCH_3_ | 0.95(d) |
|  | γ'CH_3_ | 0.82(d) |
| 3-Hydroxyphenylacetate | C_2_H,Ring | 6.79(m) |
|  | C_4_H,Ring | 6.78(m) |
|  | C_5_H,Ring | 7.24(t) |
|  | C_6_H,Ring | 6.85(m) |
|  | αCH2 | 3.47(s) |
| 3-Indoxylsulfate | C_2_H | 7.36(s) |
|  | C_4_H | 7.71(m) |
|  | C_5_H | 7.18(m) |
|  | C_6_H | 7.26(m) |
|  | C_7_H | 7.51(m) |
| 3-Phenyllactate | αCH | 4.26(dd) |
|  | βCH_2_ | 3.09(dd),2.87(dd) |
|  | C_2,6_H | 7.31(m) |
|  | C_3,5_H | 7.37(m) |
|  | C_4_H | 7.30(m) |
| 4-Hydroxyphenylacetate | C_2,6_H,Ring | 7.15(m) |
|  | C_3,5_H,Ring | 6.86(m) |
|  | αCH_2_ | 3.44(s) |
| Acetate | βCH_3_ | 1.90(s) |
| Acetoacetate | CH_2_ | 3.45(s) |
|  | CH_3_ | 2.28(s) |
| Acetone | CH_3_ | 2.22(s) |
| Alanine | βCH_3_ | 1.46(d,7.3Hz) |
|  | CH | 3.77(m) |
| Allantoin | NH,Ring | 6.06(s,br) |
|  | CH,Ring | 5.38(s) |
| Arginine | αCH | 3.76(t) |
|  | βCH_2_ | 1.88(m),1.92(m) |
|  | γCH_2_ | 1.64(m),1.72(m) |
|  | δCH_2_ | 3.23(t,9.2Hz) |
| Benzoate | C_2,6_H,Ring | 7.86(m) |
|  | C_3,5_H,Ring | 7.55(m) |
|  | C_4_H,Ring | 7.47(m) |
| Betaine | N(CH_3_)_3_ | 3.25(s) |
|  | CH_2_ | 3.89(s) |
| Carnitine | N(CH_3_)_3_ | 3.22(s) |
|  | αCH_2_ | 3.42(m) |
|  | βCH | 4.56(m) |
|  | γCH_2_ | 2.41(m) |
| Choline | N(CH_3_)_3_ | 3.19(s) |
|  | αCH_2_ | 4.07(m) |
| Citrate | βCH_2_ | 2.52(d,16.1Hz),2.68(d,16.2Hz) |
| Creatine | N-CH_3_ | 3.91(s) |
|  | CH_2_ | 3.02(s) |
| Creatine phosphate | N-CH_3_ | 3.94(s) |
|  | CH_2_ | 3.03(s) |
| Creatinine | N-CH_3_ | 4.03(s) |
|  | CH_2_ | 3.03(s) |
| Cytosine | C_5_H,Ring | 5.97(d,7.0 Hz) |
|  | C_6_H,Ring | 7.50(d,7.0 Hz) |
| Dimethyl sulfone | CH_3_ | 3.14(s) |
| Dimethylamine | CH_3_ | 2.73(s) |
| Ethanol | CH_3_ | 1.17(t,7.1Hz) |
|  | CH_2_ | 3.64(q,7.1Hz) |
| Formate | HCOO' | 8.45(s) |
| Fucose | C_1_H | 5.27(d),5.20(d) |
| Fumarate | CH= | 6.52(s) |
| Galactose | C_1_H | 5.25(d,3.6Hz),4.58(d,8.0Hz) |
| Glucose | C_1_H | 5.22(d,3.7Hz),4.64(d,8.0Hz) |
|  | C_2_H | 3.23(dd,9.5,8.2Hz), |
|  |  | 3.53(dd,10.0,3.8Hz) |
|  | C_3_H | 3.48(t,9.2Hz),3.70(t,9.3Hz) |
|  | C_4_H | 3.39(t,9.3Hz),3.40(t,9.6Hz) |
|  | C_5_H | 3.46(m),3.82(m) |
|  | C_6_H | 3.72(dd,11.4,5.6Hz),3.76(dd,12.1,5.2Hz) |
|  |  | ,3.89(dd,12.3,2.2Hz),3.84(m) |
| Glutamine | αCH | 3.76(t) |
|  | βCH | 2.10(m),2.14(m) |
|  | γCH_2_ | 2.44(m) |
| Glycerol | CH_2_ | 3.65(m),3.56(m) |
|  | CH | 3.78(m) |
| Glycine | αCH_2_ | 3.54(s) |
| Glycolate | αCH_2_ | 3.94(s) |
| Hippurate | C_2,6_H,Ring | 7.54(m) |
|  | C_3,5_H,Ring | 7.63(m) |
|  | C_4_H,Ring | 7.83(m) |
|  | αCH_2_ | 3.96(d) |
| Hypoxanthine | C_2_H,Ring | 8.18(s) |
|  | C_8_H,Ring | 8.20(s) |
| Inosine | C_1_H | 8.30(s) |
|  | C_4_H | 8.21(s) |
|  | C_6_H | 6.07(d,5.8Hz) |
|  | C_7_H | 4.77(t) |
|  | C_8_H | 4.42(m) |
|  | C_9_H | 4.27(m) |
|  | C_10_H | 3.90(dd),3.83(dd) |
| Isobutyrate | CH_3_ | 1.06(d,7.1Hz) |
|  | CH | 2.38(m) |
| Isoleucine | αCH | 3.66(d) |
|  | βCH | 1.97(m) |
|  | γCH_2_ | 1.25(m),1.45(m) |
|  | γ'CH_3_ | 1.00(d,7.2Hz) |
|  | δCH_3_ | 0.92(t,7.4Hz) |
| Lactate | βCH_3_ | 1.34(d,6.9Hz)) |
|  | αCH | 4.11(q,6.9Hz) |
| Mannitol | C_1,6_H | 3.87(dd) |
| Methanol | CH_3_ | 3.34(s) |
| Methionine | αCH | 3.81(m) |
|  | βCH_2_ | 2.09(m) |
|  | γCH_2_ | 2.64(t,7.7Hz) |
|  | S-CH_3_ | 2.12(s) |
| Methylamine | CH_3_ | 2.60(s) |
| Methylsuccinate | CH_3_ | 1.06(d,7.0Hz) |
|  | αCH | 2.61(m) |
|  | βCH_2_ | 2.11(dd),2.51(dd) |
| N,N-Dimethylglycine | CH_3_ | 2.91(s) |
|  | CH_2_ | 3.71(s) |
| N-Acetylglutamate | CH_3_ | 2.02(s) |
|  | αCH | 4.09(m) |
|  | βCH_2_ | 1.86(m),2.04(m) |
|  | γCH_2_ | 2.22(t) |
| N-Acetylglycine | CH_3_ | 2.02(s) |
|  | CH_3_ | 3.74(d) |
| N-Phenylacetylglycine | C_2,6_H,Ring | 7.54(m) |
|  | C_3,5_H,Ring | 7.34(m) |
|  | C_4_H,Ring | 7.35(m) |
|  | αCH_2_ | 3.67(m) |
|  | δCH_2_ | 3.75(d) |
| Nicotinamide N-oxide | C_2_H | 8.73(m) |
|  | C_4_H | 8.11(m) |
|  | C_5_H | 7.73(m) |
|  | C_6_H | 8.48(m) |
| Nicotinurate | C_2_H | 8.93(m) |
|  | C_4_H | 8.24(m) |
|  | C_5_H | 7.51(m) |
|  | C_6_H | 8.60(m) |
| O-Acetylcarnitine | NCH_3_ | 3.18(s) |
| O-Phosphocholine | NCH_3_ | 3.21(s) |
|  | αCH_2_ | 4.15(m) |
|  | βCH_2_ | 3.58(m) |
| Phenylalanine | C_2,6_H,Ring | 7.31(m) |
|  | C_4_H,Ring | 7.38(m) |
|  | C_3,5_H,Ring | 7.41(m) |
|  | αCH | 3.99(dd) |
|  | βCH_2_ | 3.11(dd),3.27(dd) |
| Proline | C_4_H,Ring | 1.97(m),2.02(m) |
|  | C_3_H,Ring | 2.06(m),2.34(m) |
|  | C_5_H,Ring | 3.32(m),3.41(m) |
|  | C_2_H,Ring | 4.12(m) |
| Pyruvate | CH_3_ | 2.36(s) |
| Sarcosine | αCH_2_ | 3.60(s) |
|  | γCH_3_ | 2.73(s) |
| Succinate | (α, β)CH_2_ | 2.39(s) |
| Tartrate | αCH | 4.34(s) |
| Taurine | αCH_2_ | 3.42 (t,6.7 Hz) |
|  | βCH_2_ | 3.27(t,6.7 Hz) |
| Threonine | αCH | 3.58(d,6.7Hz) |
|  | βCH | 4.25(m) |
|  | γCH_3_ | 1.30(d,6.7Hz) |
| Trigonelline | C_2_H,Ring | 9.11(s) |
|  | C_4_H,Ring | 8.82(m) |
|  | C_5_H,Ring | 8.07(m) |
|  | C_6_H,Ring | 8.84(m) |
|  | CH_3_ | 4.43(s) |
| Trimethylamine N-oxide | CH_3_ | 3.25(s) |
| Tryptophan | C_2_H,Ring | 7.31(s) |
|  | C_4_H,Ring | 7.72(m) |
|  | C_5_H,Ring | 7.17(m) |
|  | C_6_H,Ring | 7.27(m) |
|  | C_7_H,Ring | 7.53(m) |
| Tyrosine | αCH | 3.93(dd) |
|  | βCH_2_ | 3.04(dd),3.19(dd) |
|  | C_3,5_H,Ring | 6.88(d,8.5Hz) |
|  | C_2,6_H,Ring | 7.17(d,8.5Hz) |
| Urea | NH_2_ | 5.80(s,br) |
| Urocanate | C_2_H,Ring | 7.82(s) |
|  | C_5_H,Ring | 7.38(s) |
|  | αCH | 6.40(d,15.8) |
|  | βCH | 7.29(d) |
| Valine | αCH | 3.60(d) |
|  | βCH | 2.26(m) |
| Xanthosine | C_1_H,Ring | 5.85(d) |
| Xylose | C_1_H | 5.19(d),4.57(d) |
| cis-Aconitate | CH | 5.74(t) |
|  | CH_2_ | 3.12(d,1.5 Hz) |
| trans-Aconitate | CH_2_ | 3.43(s) |
|  | CH | 6.59(s) |
| π-Methylhistidine | C_2_H,Ring | 7.80(s) |
|  | C_4_H,Ring | 6.99(s) |

s=singlet; d=doublet; dd=double doublet; t=triplet; q=quartet; m=multiplet.
