# Supplementary material for: A 1H NMR-Based Metabonomic Investigation of Time-Related Metabolic Trajectories of the Plasma, Urine and Liver Extracts of Hyperlipidemic Hamsters
Source: PLoS One. 2013 Jun 26;8(6):e66786. doi: 10.1371/journal.pone.0066786 (PMC3694122; doi:10.1371/journal.pone.0066786)
Supplement: Table S6 — Quantitative metabolites in liver aqueous extracts and corresponding 1H Chemical shift assignment used for quantification. (DOCX) [file pone.0066786.s006.docx]

| **Table S6.** Quantitative metabolites in liver aqueous extracts and corresponding ^1^H Chemical shift assignment used for quantification | | |
| --- | --- | --- |
| **Metabolites** | **Moieties** | **δ^1^H(ppm)and multiplicity** |
| 2-Aminobutyrate | αCH | 3.71(m) |
|  | βCH_2_ | 1.89(m) |
|  | γCH_3_ | 0.97(t) |
| 2-Hydroxybutyrate | αCH | 3.99(dd,6.6,4.5Hz) |
|  | βCH_2_ | 1.64(m),1.73(m) |
|  | γCH_3_ | 0.89(t,7.5Hz) |
| 2-Hydroxyisobutyrate | CH_3_ | 1.36(s) |
| 2-Hydroxyisovalerate | αCH | 3.84(d) |
|  | βCH | 2.01(m) |
|  | γCH_3_ | 0.95(d) |
|  | γ'CH_3_ | 0.82(d) |
| 3-Hydroxybutyrate | αCH | 4.15(m) |
|  | βCH_2_ | 2.29(dd,14.4,6.3),2.39(m) |
|  | γCH_3_ | 1.19(d,6.3Hz) |
| 3-Hydroxyisovalerate | CH_3_ | 1.26(s) |
|  | CH_2_ | 2.35(s) |
| 3-Indoxylsulfate | C_2_H | 7.36(s) |
|  | C_4_H | 7.71(m) |
|  | C_5_H | 7.18(m) |
|  | C_6_H | 7.26(m) |
|  | C_7_H | 7.51(m) |
| 3-Phenyllactate | αCH | 4.26(dd) |
|  | βCH_2_ | 3.09(dd),2.87(dd) |
|  | C_2,6_H | 7.31(m) |
|  | C_3,5_H | 7.37(m) |
|  | C_4_H | 7.30(m) |
| Acetate | βCH_3_ | 1.90(s) |
| Acetone | CH_3_ | 2.22(s) |
| Adenine | Ring protons | 8.16(s) |
| Adenosine | Ring protons | 8.33(s),8.25(s),6.06(d) |
| Alanine | βCH_3_ | 1.47(d,7.2Hz) |
| Arginine | αCH | 3.76(t) |
|  | βCH_2_ | 1.88(m),1.92(m) |
|  | γCH_2_ | 1.64(m),1.72(m) |
|  | δCH_2_ | 3.23(t,9.2Hz) |
| Aspartate | αCH | 3.89(dd) |
|  | βCH_2_ | 2.66(dd,8.8,17.4Hz), |
|  |  | 2.80(dd,17.4,3.8Hz) |
| Betaine | N(CH_3_)_3_ | 3.25(s) |
|  | CH_2_ | 3.89(s) |
| Cadaverine | αCH_2_ | 3.01(t) |
|  | βCH_2_ | 1.71(m) |
|  | γCH_2_ | 1.46(m) |
| Carnitine | N(CH_3_)_3_ | 3.22(s) |
|  | αCH_2_ | 3.42(m) |
|  | βCH | 4.56(m) |
|  | γCH_2_ | 2.41(m) |
| Choline | N(CH_3_)_3_ | 3.19(s) |
|  | αCH_2_ | 4.07(m) |
|  | βCH_2_ | 3.51(m) |
| Citrate | βCH_2_ | 2.52(d,16.1Hz),2.68(d,16.2Hz) |
| Creatine | N-CH_3_ | 3.91(s) |
|  | CH_2_ | 3.02(s) |
| Dimethyl sulfone | CH_3_ | 3.14(s) |
| Dimethylamine | CH_3_ | 2.73(s) |
| Ethanol | CH_3_ | 1.17(t,7.1Hz) |
|  | CH_2_ | 3.64(q,7.1Hz) |
| Ethylene glycol | CH_2_ | 3.66(s) |
| Formate | HCOO' | 8.45(s) |
| Fumarate | CH= | 6.52(s) |
| Glucose | C_1_H | 5.22(d,3.7Hz),4.64(d,8.0Hz) |
|  | C_2_H | 3.23(dd,9.5,8.2Hz), |
|  |  | 3.53(dd,10.0,3.8Hz) |
|  | C_3_H | 3.48(t,9.2Hz),3.70(t,9.3Hz) |
|  | C_4_H | 3.39(t,9.3Hz),3.40(t,9.6Hz) |
|  | C_5_H | 3.46(m),3.82(m) |
|  | C_6_H | 3.72(dd,11.4,5.6Hz),3.76(dd,12.1,5.2Hz) |
|  |  | ,3.89(dd,12.3,2.2Hz),3.84(m) |
| Glutamate | αCH | 3.75(dd) |
|  | βCH | 2.04(m),2.12(m) |
|  | γCH_2_ | 2.34(m) |
| Glutamine | αCH | 3.76(t) |
|  | βCH | 2.10(m),2.14(m) |
|  | γCH_2_ | 2.44(m) |
| Glutathione | αCH2 | 4.50(m) |
|  | α'CH | 3.77(m) |
|  | β'CH2 | 2.16(m) |
|  | γ'CH2 | 2.54(m) |
|  | β''CH2 | 2.94(m) |
| Glycerol | CH_2_ | 3.65(m),3.56(m) |
|  | CH | 3.78(m) |
| Glycine | αCH_2_ | 3.54(s) |
| Inosine | C_1_H | 8.30(s) |
|  | C_4_H | 8.21(s) |
|  | C_6_H | 6.07(d,5.8Hz) |
|  | C_7_H | 4.77(t) |
|  | C_8_H | 4.42(m) |
|  | C_9_H | 4.27(m) |
|  | C_10_H | 3.90(dd),3.83(dd) |
| Isoleucine | αCH | 3.66(d) |
|  | βCH | 1.97(m) |
|  | γCH_2_ | 1.25(m),1.45(m) |
|  | γ'CH_3_ | 1.00(d,7.2Hz) |
|  | δCH_3_ | 0.92(t,7.4Hz) |
| Isopropanol | CH_3_ | 1.16(d,6.2Hz) |
|  | CH | 4.01(m) |
| Lactate | βCH_3_ | 1.34(d,6.9Hz)) |
|  | αCH | 4.11(q,6.9Hz) |
| Leucine | αCH_2_ | 3.73(m) |
|  | βCH_2_ | 1.72(m) |
|  | γCH | 1.68(m) |
|  | δCH_3_ | 0.96(d) |
| Malate | αCH | 2.35(dd,15.4,10.2Hz), |
|  |  | 2.66(15.4,3.1Hz) |
|  | βCH_2_ | 4.30(dd) |
| Methanol | CH_3_ | 3.34(s) |
| Methylsuccinate | CH_3_ | 1.06(d,7.0Hz) |
|  | αCH | 2.61(m) |
|  | βCH_2_ | 2.11(dd),2.51(dd) |
| N,N-Dimethylglycine | CH_3_ | 2.91(s) |
|  | CH_2_ | 3.71(s) |
| N-Acetylglutamate | CH_3_ | 2.02(s) |
|  | αCH | 4.09(m) |
|  | βCH_2_ | 1.86(m),2.04(m) |
|  | γCH_2_ | 2.22(t) |
| N-Acetylglycine | CH_3_ | 2.02(s) |
|  | CH_3_ | 3.74(d) |
| NAD+ | Ring protons | 9.32(s),9.14(d,6.4Hz),8.82(m),8.42(s), |
|  |  | 8.20(dd),8.16(s),6.08(d),6.03(d,5.9Hz) |
| NADP+ | Ring protons | 9.28(s),9.10(d),8.81(m),8.41(s), |
|  |  | 8.18(dd),8.13(s),6.10(d),6.03(d) |
| Nicotinurate | Ring protons | 8.92(dd),8.70(dd,5.0,1.6Hz), |
|  |  | 8.23(dt),7.58(m) |
|  | αCH_2_ | 3.98(m) |
| O-Acetylcarnitine | NCH_3_ | 3.18(s) |
| O-Phosphocholine | NCH_3_ | 3.21(s) |
|  | αCH_2_ | 4.15(m) |
|  | βCH_2_ | 3.58(m) |
| Ornithine | αCH | 3.77(t) |
|  | βCH_2_ | 1.92(m) |
|  | γCH_2_ | 1.82(m),1.73(m) |
|  | δCH_3_ | 3.04(t,7.6Hz) |
| Succinate | (α, β)CH_2_ | 2.39(s) |
| Taurine | N-CH_2_ | 3.24(t) |
|  | S-CH_2_ | 3.40(t) |
| Tyrosine | αCH | 3.93(dd) |
|  | βCH_2_ | 3.04(dd),3.19(dd) |
|  | C_3,5_H,Ring | 6.88(d,8.5Hz) |
|  | C_2,6_H,Ring | 7.17(d,8.5Hz) |
| UDP-galactose | Ring protons | 7.94(d),5.96(d),5.63(dd) |
| UDP-glucose | Ring protons | 7.94(d),5.97(d),5.60(dd) |
| UDP-glucuronate | Ring protons | 7.94(d),5.97(d),5.61(dd) |
| Uridine | Ring protons | 7.85(d,8.1Hz),5.90(d,4.5Hz), |
|  |  | 5.88(d,8.1Hz) |
| Valine | αCH | 3.60(d) |
|  | βCH | 2.26(m) |
| s=singlet; d=doublet; dd=double doublet; t=triplet; q=quartet; m=multiplet. | |  |
